# Development of indicators to measure health system capacity for quality abortion care in 10 countries: a rapid assessment of a measurement framework and indicators

**DOI:** 10.1136/bmjph-2023-000401

**Published:** 2024-05-06

**Authors:** Heidi Bart Johnston, Ulrika Rehnstrom Loi, Mohamed Ali, Katy Footman, Ghislaine Glitho Alinsato, Eman Aly, Asmani Chilanga, Shikha Bansal, Laurence Codjia, Fahdi Dkhimi, Sithembile Dlamini-Nqeketo, Hayfa Elamin, Dina Gbenou, Karima Gholbzouri, Lisa Hedman, Nilmini Hemachandra, Yelmali Hien, Md Khurshid Alam Hyder, Theopista John, Amrita Kansal, Priya Karna, Laurence Läser, Antonella Lavelanet, Belete Mihretu, Pamela Amaka Onyiah, Leopold Ouedraogo, Sikander Qais, Ellen Thom, Meera Upadhyay, Qudsia Uzma, Souleymane Zan, Bela Ganatra

**Affiliations:** 1 UNDP-UNFPA-UNICEF-WHO-World Bank Special Programme of Research, Development and Research Training in Human Reproduction (HRP), Department of Sexual and Reproductive Health and Research, World Health Organization, Geneva, Switzerland; 2 World Health Organization Country Office for Benin, Cotonou, Benin; 3 World Health Organisation Regional Office for the Eastern Mediterranean, Cairo, Egypt; 4 Formerly with World Health Organization Africa Regional Office, Ouagadougou, Burkina Faso; 5 World Health Organisation Country Office for India, New Delhi, Delhi, India; 6 Department of Health Workforce, World Health Organization, Geneva, Switzerland; 7 Department of Health Systems Governance and Financing, World Health Organization, Geneva, Switzerland; 8 World Health Organization Country Office for South Africa, Pretoria, South Africa; 9 World Health Organization Africa Regional Office, Harare, Zimbabwe; 10 Formerly with World Health Organization Country Office for Burkina Faso, Ouagadougou, Burkina Faso; 11 Special initiatives, Access to Medicines and Health Products, World Health Organization, Geneva, Switzerland; 12 World Health Organization Country Office for Myanmar, Yangon, Yangon Region, Myanmar; 13 World Health Organization Country Office for Burkina Faso, Ouagadougou, Burkina Faso; 14 World Health Organization Country Office for Nepal, Kathmandu, Nepal; 15 World Health Organization Country Office for Rwanda, Kigali, Rwanda; 16 Formerly with World Health Organization Country Office for Nepal, Kathmandu, Nepal; 17 World Health Organization Regional Office for Africa, Brazzaville, Congo; 18 Formerly with World Health Organization Eastern Mediterranean Regional Office, Cairo, Egypt; 19 World Health Organization Country Office for Pakistan, Islamabad, Pakistan; 20 World Health Organization South East Asia Regional Office, New Delhi, India

**Keywords:** Public Health, methods, Public Health Practice

## Abstract

**Introduction:**

A significant gap exists in the availability of indicators and tools to monitor health system capacity for quality abortion care at input and process levels. In this paper, we describe the process and results of developing and assessing indicators to monitor health system capacity strengthening for quality abortion care.

**Methods:**

As part of a 4-year (2019–2022) multicountry project focused on preventing unsafe abortion using a health system strengthening approach in 10 countries, we developed a monitoring framework with indicators and metadata. Through an internal consultative process, we identified a structured list of operational health system capacity indicators for abortion. After implementing the indicators for baseline and annual project monitoring, project staff from 10 teams assessed each indicator using 4 criteria: validity, feasibility, usefulness and importance.

**Results:**

We identified 30 indicators aligning with 5 of the 6 WHO health system building blocks (excluding service delivery): 6 indicators in leadership and governance, 5 in health workforce, 6 in health information, 8 in access to medicines and health products and 5 in health financing. In our assessment of indicators, average scores against the predetermined criteria were lowest for feasibility (7.7 out of 10) compared with importance (8.5), usefulness (8.9) and validity (9.3). Assessors highlighted the need for fewer and less complex indicators, simplified language, clearer benchmarks, for indicators to be abortion-specific, less subjective and for future frameworks to also include service delivery and research and innovation.

**Conclusion:**

We used 30 indicators to monitor health system capacity for quality abortion care in 10 countries and gathered critical feedback that can be used to further strengthen the set of indicators in future work. Establishing core input and process indicators will be critical to inform and support evidence-based policy and programme improvements for quality abortion care.

**WHAT IS ALREADY KNOWN ON THIS TOPIC**

- A recent scoping review highlighted a lack of indicators that can be used to monitor health system capacity to provide quality abortion care.

**WHAT THIS STUDY ADDS**

- We designed, implemented, monitored and gathered critical feedback on 30 indicators to measure health system capacity to provide quality abortion care.

**HOW THIS STUDY MIGHT AFFECT RESEARCH, PRACTICE OR POLICY**

- We have progressed towards identifying appropriate domains and indicators for monitoring health system capacity to provide quality abortion care. Additionally, we have outlined further work that needs to be done before the indicators can be recommended. Establishing indicators for measuring health system capacity to provide quality abortion care is key to motivating evidence-based action and accountability in sexual and reproductive health and rights and thus to meeting Sustainable Development Goal commitments.

## Introduction

 Strong health systems are required to achieve Sustainable Development Goal targets 3.1, 3.7 and 5.6, which ensure sexual and reproductive health services and rights. However, there are severe weaknesses in health system capacity to deliver quality abortion care in many countries, such as low facility readiness or poor availability of essential equipment and skills.^1 2^

Effective monitoring and evaluation is essential to inform evidence-based programme design, but monitoring of health system capacity to provide quality abortion care is lacking. Monitoring health system performance requires an assessment of inputs and processes (eg, governance), outputs (eg, service readiness), outcomes (eg, intervention coverage) and impact (eg, health outcomes).^3^ A recent extensive scoping review identified that most abortion indicators focus on outputs, outcomes and impact (eg, access, availability and physical health),^4^ and do not pay adequate attention to inputs and processes, although these are critical elements of health system performance.^3 5^ The scoping review highlighted the need to develop and test new indicators for monitoring health system capacity to provide quality abortion care.^4^

General health system inputs and processes are well-defined. The WHO health system building blocks are essentially inputs and processes, and these include leadership and governance, health workforce, health information systems, medical products and technologies, health financing and service delivery.^6^

Although existing health system framework indicators can monitor overall health system capacity,^3 6 7^ they are not sufficient to effectively monitor health system capacity to deliver quality abortion care, as abortion is often sidelined or excluded from mainstream health education, planning, service provision and financing.^8 9^ For example, health worker density and distribution indicators do not tell us whether there is adequate density and distribution of health workers that have essential competencies or legal authorisation to provide abortion care. For stigmatised or neglected areas of health, it is particularly critical for disease-specific or topic-specific indicators to be integrated into common platforms, data process and management systems.^10^ Abortion has often historically been excluded from monitoring indicators and initiatives^11 12^ and even monitoring frameworks for sexual and reproductive health (SRH) often exclude abortion.^13 14^

A significant gap therefore exists in the availability of indicators and tools to monitor health system capacity to provide quality abortion care at the input and process levels. In this paper, we describe the process and results of developing input and process level indicators designed to measure health system capacity to provide quality abortion care. We define quality abortion care as care that is effective, efficient, accessible, acceptable, equitable and safe and includes abortion care, management of complications after an abortion and services after abortion such as contraceptive services and linkages to other relevant services.^15^

## Methods

We describe the process of developing indicators to monitor a 4-year project focusing on health system strengthening to prevent unsafe abortion in ten countries. This is followed by a descriptive analysis of an internal evaluation of the indicators.

### Project structure

In 2019, we formed as a team of WHO technical staff to implement a 4-year (2019–2022) multicountry, multiregion project of the WHO and the United Nations Development Programme–United Nations Population Fund–United Nations Children’s Fund–WHO–World Bank Special Programme of Research, Development and Research Training in Human Reproduction. The project focused on strengthening and supporting countries’ national health systems to have the structures necessary to deliver accessible, affordable and quality abortion services within a country’s universal health coverage framework. Health system strengthening project activities included efforts to strengthen policies, norms, standards, guidelines, curricula, monitoring tools, medicines procurement, health financing mechanisms, and other administrative tools relevant for quality abortion care. Service delivery activities were beyond the scope of the project and were not included. Countries were selected based on the need to strengthen the health system to prevent unsafe abortion, high level interest to prevent unsafe abortion within the Ministry of Health, support from the executive branch of government and geographic diversity. In total, 10 countries were included in the project across 4 WHO regions: Benin, Burkina Faso, Rwanda, South Africa, Sierra Leone, India, Nepal, Myanmar, Lao People’s Democratic Republic and Pakistan.

The project team comprised 25 WHO technical staff based in the ten country offices, 3 regional offices (Africa, Eastern Mediterranean and South-East Asia) and headquarters, based in Geneva, Switzerland. As a whole, the team included technical staff from the areas of abortion, sexual and reproductive health and rights (SRHR), reproductive health policy, health workforce, data and analytics, health information systems, access to medicines and health products, and health financing. Members of this team jointly developed the indicators. Team members based in the project countries—who tended to have broad SRH programme implementation expertise—implemented the indicators as a baseline and for end of year monitoring, accessing information from available data sources. The entire team was invited to participate in the indicator assessment described in this paper.

### Monitoring framework and indicator development and implementation

To monitor project progress in the focus countries, the team initially tried to identify existing input and process health system indicators for abortion through a review of international health-related indicator databases including those of Measure Evaluation, Demographic and Health Surveys, and Multiple Indicator Cluster Surveys. Subject matter specialists in areas of abortion law and policy, health workforce, health information systems, access to medicines and products, health financing and SRH programme implementation, conducted a review of indicators within their area of technical expertise. When it became clear that no existing set of indicators measured health system capacity for providing quality abortion care, we recognised the need to develop our own set of indicators. As a team, we convened regularly in 2019–2020 to develop and internally validate a set of indicators using guiding principles which we developed for the purpose, listed below. In some countries, programme managers within Ministries of Health were also involved in these discussions.

We agreed to use the WHO health system building block structure^6^ to define indicator domains because the building blocks are essential determinants of health system performance and are widely appreciated as country-focused, supportive of country needs, while also providing a basis for global monitoring.^3 16 17^ We included five of the six health system building block domains (leadership and governance, health workforce, health information systems, medical products and technologies, health financing) and excluded the service delivery domain. The included building block domains—representing inputs and processes which are generally in the control of government agencies—align with most of the health system strengthening activities of the project. Indicators within each domain focus on inputs and processes that need to be in place to support quality abortion service delivery whether the services are provided by state or non-state actors.

In most cases, the indicators proposed were based on existing indicators already used in WHO national and global monitoring for other health topics. These were then altered to be specific to abortion care. We developed and included abortion indicators based on the following guiding principles. Indicators should:

- Be measurable with information from existing documents, data routinely collected through country health information systems, WHO monitoring systems or similar administrative sources.
- Align with existing, validated indicators to ensure similarity with those used for other health topics.
- Represent an important component of health system capacity to prevent unsafe abortion and support quality abortion care.
- Be useful for programme planning or programme improvement within and beyond the project.
- Align with existing WHO strategy and guideline documents,^18^,^27^ including WHO’s 13th General Programme of Work.^28^

We then deliberated and updated the set of proposed indicators in multiple virtual meetings and in one in-person consultation with the project team. To support the measurement of each indicator consistently across countries and over time, we developed indicator metadata, which were reviewed by the project team. Metadata included the indicator name, rationale for inclusion, definition, method of calculation (when needed), preferred data sources, alternative data sources, limitations and references supporting the indicator (online supplemental appendix 1).

To monitor project progress, staff members on the project team based in the 10 WHO country project offices measured the full set of indicators at baseline and annual follow-ups using a pretested monitoring tool (online supplemental appendix 2), which facilitated comparability of results over time and across countries. Country office-based technical officers collected data from administrative sources such as national policy documents, clinical training curricula, health management information system survey instruments, financial tracking systems and other relevant sources. Country office-based technical officers documented the evidence sources in the data collection tool. If available, electronic versions of evidence sources were submitted with the monitoring results. Monitoring results were used to show high level project performance at the country level.

### Assessment of indicators

After conducting a baseline and one or more rounds of annual monitoring, we conducted an assessment of the indicators. Our assessment process and criteria were informed by those used in other indicator prioritisation activities (eg, WHO 100 core indicators,^29^ the Ending Preventable Maternal Mortality monitoring framework development^30^). We developed and implemented an assessment tool to collect feedback on the indicators. For each of the indicators, assessors were asked to score quality using four criteria: validity, utility, feasibility and importance. Practical, applied definitions for each criterion were also developed. We defined *validity* as ‘the indicator makes sense, the definition is clear and the indicator was applicable to the context’. We defined *feasibility* as ‘the necessary information to construct the indicator was accessible and easy to collect once the correct source was identified’. We defined *utility* as ‘the information collected was useful for programme planning or programme improvement’. We defined *importance* as ‘the information represents an important component of health system capacity to prevent unsafe abortion and provide safe abortion’.

In 2022, the structured assessment of the indicators was completed by members of the project team. Members received an assessment tool (online supplemental appendix 3) and were asked to provide feedback on each of the indicators individually and as an entire indicator set. Some assessment tools were completed by multiple individuals from a country or regional office working together as a team, while some were completed by an individual working alone. Feedback was provided by project team members from 7 out of the 10 country offices (Benin, Burkina Faso, India, Nepal, Pakistan, Rwanda, Sierra Leone), 2 out of the 3 regional offices (Eastern Mediterranean, South-East Asia) and the headquarters team access to medicines and health products.

For each indicator, assessors noted whether they thought the indicator met the four criteria used for indicator assessment (validity, utility, feasibility and importance) and gave a score of 0 (criteria not met) or 1 (met). The criteria definitions were included in the feedback tool, to ensure the criteria were understood in the same way by each assessor. In addition, assessors were encouraged to provide text comments to explain their assessment of each indicator and to provide comments on the indicator set as a whole.

Following the assessment, the full project team held a virtual meeting at which assessment results were presented, discussed and internally validated.

We summarised the scored and text results from the structured evaluation. To generate summary scores, we summed scores for each indicator for each criterion to provide a total, which could range from 0 to 10, as 10 assessment teams completed the evaluation. We also calculated mean scores for each indicator criterion across all indicators and for each domain.

### Patient and public involvement

The authors declare that no patients were involved in this research.

## Results

A total of 30 new indicators were developed across the 5 health system building block domains (table 1). Key definitions, data source suggestions and reference documents are included in the metadata document in online supplemental appendix 1.

**Table 1:** Indicators by building block, with average scores and assessor feedback on the limitations of each indicator

Domain	#	Indicator	Total score (n=10)				Feedback on limitations of indicators
			Valid	Feasible	Useful	Important	
Leadership and governance	1.1	Decreasing unsafe abortion is part of national strategy, plan or similar (eg, for maternal and neonatal health, reproductive health, or similar).	10	9	10	8	National strategies may include maternal mortality reduction without specifying unsafe abortion. The indicator could be adapted to include ‘Decreasing unsafe abortion or reducing maternal mortality is part of…’, though others felt it was important for abortion to be specifically mentioned.
	1.2	SRHR integrated into country cooperation strategy and other relevant national strategic documents/roadmaps (eg, UNDAF).	10	10	10	10	No additional comments were provided.
	1.3	MoH has SRHR steering group or coordination mechanism operated with WHO participation and support.	10	10	10	10	For use outside the project, the indicator could be adapted to focus on the existence of a steering group, regardless of whether it is operated with WHO support.
	1.4	Protocols for comprehensive abortion care (SA and PAC) aligned with global standards are in national medical/treatment guidelines.	10	10	10	10	No additional comments were provided, but the indicator could be amended to include FP protocols.
	1.5	Number of laws/policies/strategies/regulations/guidelines developed or updated in alignment with global or WHO SRHR guidelines.	10	10	10	9	Indicator could measure whether there is alignment, instead of number aligned, to aid interpretation. Laws/regulations could be separated from policies/strategies. Challenging to monitor due to difficulty of keeping pace with increasingly regular scientific developments.
	1.6	Descriptive assessment of extent to which tools and guidance to operationalise comprehensive abortion care (SA, PAC and FP) enabling policies/guidelines etc exist.	8	7	8	7	This indicator is subjective, and criteria are unclear, limiting comparability over time or across countries.
	Additional indicator to consider, based on assessor feedback: indicators to measure data-driven decision-making for SRHR, generation of political/high-level commitment and capacity building or orientation of policy-makers in SRHR.						
Health workforce	2.1	Existence of institutional models for assessing and monitoring staffing needs for sexual and reproductive health service delivery.	8	6	8	8	Staffing needs assessment are conducted by cadre, not by the specific functions staff serve. Indicator should specify ‘qualified’ health workers and that the institutional model must be operationalised. Indicator is very complex to track.
	2.2	Proportion of accredited education institutions for all relevant cadres with a competency-based SRHR component in curricula (inclusive of SA/PAC/FP), consistent with global normative guidance.	10	4	9	8	Complex and difficult to measure. This indicator includes three areas (SA/PAC/FP), facilities need to be assessed as ‘accredited’, training needs to be assessed as ‘competency based’ and ‘consistent with global normative guidance’. Need to specify as in-service training.
	2.3	Number of graduates in past year from accredited education institutions with a competency-based SRHR component in curricula (inclusive of SA/PAC/FP), consistent with global normative guidance—for all relevant cadres.	10	5	9	7	The critique of indicator 2.2 applies to 2.3. Also, data are lacking or located outside the Ministry of Health. Ministry of Education data access can be challenging.
	2.4	Country has system for in-service competency-based training in comprehensive abortion care, consistent with global normative guidance.	10	9	10	10	May be difficult to track with existing systems.
	2.5	Health workforce policies provide guidance to operationalise SRHR related priorities (eg, urban–rural distribution, task sharing/skill mix, community health worker utilisation, etc).	9	9	9	8	Subjective, requires narrative reporting, limiting comparability. Does not capture whether policies are implemented.
	Additional indicator to consider, based on assessor feedback: competency-based training guidelines for SRHR have been endorsed at the national level.						
Health information	3.1	List of essential SRHR indicators, including SA, PAC and FP indicators, established within the national health system.	10	10	9	10	Indicators 3.1 and 3.2 are duplicative and could be merged.
	3.2	Essential SRHR indicators, including SA, PAC and FP indicators, integrated into national health information system.	9	9	9	9	
	3.3	DHIS2 module for SRHR, including SA, PAC and FP indicators, integrated into national HMIS.	8	8	9	7	SRHR indicators may be included within an RMNCH module. Indicator could be for HMIS more broadly, not just DHIS2.
	3.4	Essential SRHR (SA, PAC and FP) indicator data quality periodically assessed using WHO data quality review tools.	10	6	9	9	The indicator is difficult to measure with information from existing sources. Data quality review tools are cumbersome to monitor periodically.
	3.5	Comprehensive abortion care (SA, PAC and FP) module integrated into the WHO health facility survey tool (HHFA or SARA) and/or other national monitoring platforms.	10	10	10	9	No additional comments were provided.
	3.6	HMIS SRHR data, including data on SA, PAC and FP, used for planning, budgeting or fundraising activities.	10	8	10	10	The indicator is difficult to measure with information from existing sources.
	Additional indicator to consider, based on assessor feedback: country monitors, publishes routinely and/or has functional Civil Registration and Vital Statistics and Maternal Perinatal Death Surveillance and Response systems, to effectively monitor maternal/perinatal mortality.						
Access to medicines and health products	4.1	National Essential Medicines List includes combination mifepristone and misoprostol, or misoprostol and mifepristone as separate presentations.	10	10	10	10	Specify the use for which misoprostol is included in the national essential medicines list.
	4.2	Number of combination mifepristone and misoprostol and/or misoprostol and mifepristone as separate presentations submitted for market authorisation, including through the WHO collaborative registration procedure for prequalified products.	9	6	9	8	Difficult to measure from existing sources, and less important than other medicines indicators.
	4.3	Number of medical abortion products registered (combination mifepristone and misoprostol and/or misoprostol and mifepristone as separate presentations).	10	9	9	10	Can be time-consuming to gather data. Could measure whether each medicine is registered, rather than number registered.
	4.4	Pharmacovigilance system in place to monitor combination mifepristone and misoprostol and/or misoprostol and mifepristone as separate presentations.	10	7	9	10	Difficult to measure from existing sources. Vertical programmes may not be integrated in pharmacovigilance systems. Pharmacovigilance system should not just be ‘in place’ but also ‘set up’.
	4.5	Combination mifepristone and misoprostol and/or misoprostol and mifepristone as separate presentations, are on national procurement lists, including tenders or other relevant documents.	10	9	10	9	Difficult to measure with information from existing sources. Procurement may not happen at national level.
	4.6	Combination mifepristone and misoprostol and/or misoprostol and mifepristone as separate presentations, procured in past 24 months via recognised procurement agents that serve the public sector.	9	9	9	9	No additional comments were provided.
	4.7	Forecasting tools for safe abortion essential medicines and products improved to align with national service capacity and to capture relevant information for national/regional market.	9	4	7	8	Some teams in countries with restrictive abortion laws found this indicator not relevant. This is a complicated indicator, and could be clarified by replacing ‘improved’ with ‘n place’.
	4.8	Number of regulators participating in prequalification trainings, observations, fellowships and other efforts.	7	7	7	6	Difficult to measure at the country level, not very relevant to medicine availability and not SRHR specific.
	Additional indicator to consider, based on assessor feedback: availability of surgical abortion technologies, availability of postabortion contraception.						
Health financing	5.1	Essential SRH services have been assessed for inclusion in the benefit package as part of a systematic process including criteria on economic evidence and budget impact/costs.	10	8	8	9	Complex and difficult to interpret.
	5.2	Number of health financing arrangements that have introduced new SRH essential services (including SA, PAC and FP) into their benefits package.	9	8	9	8	Complex and difficult to interpret.
	5.3	Number of health financing instruments that have critically reviewed and adjusted their purchasing modalities—for example, benefits specification including cost-sharing, payment methods, provider contracts—to boost service delivery of SRH essential services.	6	3	6	5	Complex, difficult to interpret and understood by some assessors as duplicative of 5.2. These are not intended as indicators for cross-country comparison, but rather descriptions of critical features of the resource allocation system in a given country.
	5.4	Results of analysis of demand configuration and constraints to SRH essential services assessed and factored into health financing work.	8	4	8	7	Complex and difficult to interpret and understood by some assessors as duplicative of 5.2. This could be considered in the governance rather than health financing domain.
	5.5	Public and external spending on reproductive health tracked.	9	7	8	8	Indicator was poorly understood and difficult to track in some countries. The current System of Health Accounts has a module to track reproductive health but does not specify the level of spending on CAC.
Average score			9.3	7.7	8.9	8.5	

Shading is used to illustrate higher (darker green) and lower (lighter green) average scores.

CACcomprehensive abortion careFPfamily planningHHFAharmonised health facility assessment HMIShealth management information systemMoHMinistry of HealthPACpostabortion careRMNCHReproductive, Maternal, Newborn, and Child HealthSAsafe abortionSARAservice availability and readiness assessmentSRHsexual and reproductive healthSRHRsexual and reproductive health and rightsUNDAFUnited Nations Development Assistance Framework

All indicators could be constructed with information from existing sources with the expectation that no primary data collection would be required from facilities, providers or patients and no confidential information would be collected. Depending on the indicator, existing sources could include administrative documents such as policy papers, clinical training curricula, health management information system databases and financial tracking systems. Many of the indicators were designed to facilitate collection of information separately for abortion, postabortion care and family planning, rather than for quality abortion care as a single compound unit, which enabled interpretation and identification of specific areas for improvement (online supplemental appendix 2). The newly developed indicators were specific to abortion but were similar to those used for other health topics, to ensure alignment with existing health system monitoring processes and avoid siloed abortion care monitoring processes.

Six leadership and governance indicators were created to assess the level of political commitment to SRHR, including abortion-related rights, through policies, strategies and approaches for health system governance. The five health workforce domain indicators assess the extent to which systems are in place to support adequate production, availability and distribution of health workers trained in provision of abortion-related care. The six health information system domain indicators focus on inclusion of SRH indicators in the national health information system. The eight access to medicines and health products domain indicators assess the systems in place to promote availability of quality-assured essential abortion medicines. The five health financing domain indicators assess steps toward better tracking of financing flows allocated for SRH service provision, the level of inclusion into the benefits package of major national health financing mechanisms and the way these mechanisms allocate resources to health service providers. Not all indicators were specific to abortion. However, all indicators included were understood to be representative of the enabling environment for quality abortion care.

Table 1 summarises the results from the evaluation of indicators completed by 10 assessment teams, including average scores for each criterion, and specific challenges identified by teams. Average scores were lowest for feasibility (7.7 out of 10) compared with importance (8.5), usefulness (8.9) and validity (9.3). A key feasibility issue was that some data were not available or were difficult to access, and this was reflected in high levels of missing data for some indicators (such as 2.3, 4.8 and 5.3 in table 1) during project monitoring.

Of the domains, average scores were lowest for health financing (ranging from 6.0 for feasibility to 8.4 for validity) and highest for leadership and governance (ranging from 9.0 for importance to 9.7 for validity and usefulness) (data not shown). Text feedback on the health financing indicators mostly related to the perceived complexity of these indicators, challenges with interpretation and difficulties experienced with measurement. Feasibility scores were also below average for health workforce indicators (6.6 on average), and text feedback highlighted that some of the workforce indicators (such as 2.1, 2.2 and 2.3 in table 1) were difficult to track, involved requesting information from multiple education institutions or non-health government departments such as Ministries of Education or Labour, and required multiple judgements about whether facilities are accredited, and whether training is competency based. As staffing needs assessments are conducted by cadre, rather than by the specific functions staff serve, assessors found that a lack of relevant data also made workforce indicators less feasible to measure, highlighting the need for more granular data at lower levels of the health system.

Broader feedback included the need for fewer and less complex indicators. Assessors highlighted the challenge of interpreting numbers-based indicators (such as 1.5 and 5.2 in table 1), as clear benchmarks do not exist for the desired number of policies or institutions. Assessors also noted the challenges of interpreting results from the more subjective, descriptive indicators (such as 1.6 and 2.5). During project monitoring, in some countries leadership and governance indicators (such as 1.2 and 1.4) were recorded as supportive of an enabling environment despite existing in the context of restrictive abortion policies. Some of these indicators referred to SRHR broadly rather than abortion specifically, which may explain this seeming contradiction. Some assessors felt it was important to include a greater focus on family planning, as access to family planning may be an upstream determinant of unsafe abortion.

In the feedback responding to specific domains and the set of indicators as a whole, assessors noted additional areas to potentially include. The proposed additional areas for each health system building block domain are listed in table 1. For the framework as a whole, some assessors felt that service delivery indicators and research and innovation should be included.

## Discussion

Strong health system capacity for abortion care is needed to ensure that high-quality abortion care is available and accessible. To successfully strengthen health system capacity, effective indicators are required to monitor progress, identify gaps and inform action. In this paper, we present 30 indicators to measure health system capacity for quality abortion care across five health system domains, alongside feedback reflecting the experience of implementing these indicators in 10 countries. The five health system building block domains were found to have relevance for monitoring health system capacity to provide abortion care: governance and leadership, health workforce, health information, access to medicines and health products, and health financing. Indicators that scored highly against selected criteria (validity, feasibility, usefulness, importance) may be useful for inclusion within future efforts to monitor and strengthen health system capacity for quality abortion care. The indicators have the strengths of drawing on existing data sources, not requiring primary data collection and aligning with existing health system monitoring. These characteristics reduce the resource requirements and intensiveness of the indicators and can increase motivation to use them. Many of the indicators can also inform assessments of health system capacity to support self-care for abortion, as evidence-based protocols, trained workforce and medicine registrations are essential for both provider-led and patient-led models of care.^31^ Drawing on feedback from implementation of these indicators, we identified challenges which can be remedied and areas that can be strengthened; these are discussed below.

As a set, the indicators presented in this paper have the limitation that they do not cover all six health system building block domains. The indicators were developed for a specific project and the selection process was therefore guided by the project activities we intended to monitor. We developed indicators for five of the WHO health system building blocks, but not for the area of service delivery, as the project did not include activities in this area. A recent scoping review of abortion metrics^4^ identified that among abortion care indicators, those of access, availability and provision of abortion care were the most common. This suggests service delivery indicators for abortion are already well-developed, even though they may not be commonly used.

Other key primary healthcare (PHC) operational framework levers were not included in our framework, such as engagement with communities and other stakeholders, PHC-oriented research, physical infrastructure and digital technologies for health.^32^ This point was highlighted by assessors in their feedback on the indicators, as it was noted that research and innovation indicators should be included in future work. Digital technologies have become particularly important for abortion care in recent years due to the growth in self-management of medical abortion, with telemedicine,^33^ hotlines^34^ and other digital interventions^35 36^ providing new modes to expand access to patient-centred care. Future work could develop indicators within some of these additional domains and could include some of the indicators proposed by assessors in their feedback, for example, whether competency-based training guidelines for SRHR have been endorsed at the national level in preservice education of healthcare workers. Other areas to consider in further indicator development include policies on conscientious objection to abortion care provision, policies relating to consent for abortion care based on age and marital status, the level of the health system at which abortion care and abortion medications specifically can be offered and the regulated availability of medical abortion drugs such as prescription requirements and regulatory class. By adding further detail, indicators could also assess capacity for first and second trimester abortion separately.

Assessors highlighted the need for clear indicator specification. Feedback from assessors identified the need for terms (such as competency-based training) to be clear and consistent across settings to assess whether a specified standard has been met. Some descriptive indicators, particularly those measuring the enabling environment for abortion care, were considered by assessors to be too subjective to generate comparable measurement across countries and over time or were difficult to interpret due to a lack of obvious benchmarks. Not all indicators were designed for cross-country comparison and instead were intended to describe and analyse the present situation in a country so that this information could inform future action plans (eg, 5.3: number of health financing instruments). Some indicators do not necessarily require a benchmark (eg, 1.5: number of laws and policies) as there is not an ideal number of laws and policies, but tracking the number that align with global or WHO guidelines can indicate whether the national situation is improving or declining over time. There is a need to balance the inclusion of richer, more descriptive assessments which can inform decision-making in one context, with the need for quantitative indicators that minimise subjectivity and allow tracking of change over time and between contexts. While health system monitoring is primarily useful at a national level to inform policies and programmes, cross-country comparisons can help to identify effective strategies for improving abortion access or to motivate reform. Additional precision for such comparisons could be achieved by including structured subcomponents to descriptive indicators for scoring and structured narrative reporting.

Most of our indicators were measured specifically for abortion, postabortion care and family planning. However, some indicators referred more broadly to SRHR and its integration into country cooperation strategies, health benefit package assessments and health financing instruments. Since abortion care is often excluded from efforts to improve SRHR,^13 14^ it is possible that these indicators may not be specific enough to capture whether quality abortion care has been sufficiently addressed within these mechanisms. Design of future indicators to monitor quality abortion care should consider the need for specificity, given the politicised and stigmatised nature of abortion.^37^ Additionally, although it is important to improve the visibility of health system capacity to deliver abortion care, this need must be balanced with the potential risk of siloed disease-specific or topic-specific monitoring. The indicators presented in this paper were developed to align with existing health system monitoring and tools, to facilitate integration and avoid siloed abortion care monitoring; this tension requires further consideration in future work.

Feasibility of measurement was a common challenge listed by assessors. To be used, indicators must be feasibly measurable from accessible sources. We found our indicators scored lower on feasibility than on any other criteria, and several of the indicators were only considered to be feasible by 5 or fewer out of 10 assessors. In some cases, this was because measurement was found to be complicated, involving consultation of multiple information sources. For many of the indicators, we assumed the information was available in the public domain or via communication with ministries, but this was not always the case. The feedback from assessors therefore highlighted the need for more granular data to be available at lower levels of the health system, for example, from educational institutions or health financing assessments, which could support efforts to measure topic-specific health system capacity. We intended for indicators to be measured annually in our project, but some indicators may change more rapidly than others, and future work could also consider the regularity with which each indicator should be assessed.

Health financing indicators scored particularly low in our indicator assessment, in part due to feasibility concerns but also due to perceived over-complexity. This may partially reflect the fact that most assessors were SRH specialists and did not have expertise in health financing. Similarly, some of the challenges highlighted by assessors in the domains of access to medicines and health products and health workforce could reflect a lack of expertise in these technical areas among most of the assessors. This highlights the need for collaborative working in health system strengthening, as siloed expertise can limit programme effectiveness as well as monitoring and evaluation efforts. The reliance on other sectors of government (legislation, education, finance) to measure certain indicators also highlights the need for multisectoral collaboration when monitoring and addressing health system inputs for abortion care.

Challenges with tracking the proposed health financing indicators may also highlight the lack of evidence to support health financing for quality abortion care. Health financing is a critical area for abortion care and there is a need to strengthen the capacity of SRH programmes in this area. Out-of-pocket payments are the dominant source of funding for reproductive healthcare in many countries,^38^ and economic factors can thwart care-seeking, affect the type of care sought and impact the gestational age at which care is reached.^38^ However, the fragmentation of health financing in many countries and the lack of transparency in financing data can make progress hard to measure. The 2020/2021 WHO Health Benefit Package Survey,^39^ implemented separately from the project reported on in this paper, identified services covered in health benefit packages of the largest government-financed scheme in each of 115 countries and areas. The 2020/2021 survey included abortion care for the first time, and now this indicator can be used in future work.^40^ Future indicators could also assess whether any specific conditions exist for the inclusion of abortion care in health benefit packages.^40^

As next steps, we suggest these indicators should be further reviewed and amended by the wider SRHR measurement community, to address the gaps we have identified, strengthen definitions where needed and ensure adequate balance across health system capacity domains. This process will also require consultation with broader health experts in the areas of medicines and health financing, as well as experts involved in education, labour, legislation and public finance. Indicators from project monitoring have been adopted into research projects and monitoring systems, including the Sexual and Reproductive Health Self-Care Measurement Tool.^31^ This new tool includes several of our high-scoring leadership and governance indicators to measure the enabling environment for self-managed abortion. The adoption of project indicators into research projects and tools shows the demand for indicators in this area and the urgency to move forward with next steps.

This process of developing and assessing indicators to measure health system strengthening for quality abortion care had limitations. Although a large team with broad knowledge in relevant areas developed the indicators, and there was some input from Ministries of Health at the indicator development stage, we did not then seek further validation through external review or involvement of wider stakeholders. Most implementers and assessors of the indicators were specialists in SRH and did not have technical expertise in health financing, access to medicines and health products or health workforce, which may have influenced indicator scores in these domains. We did not receive feedback from all project team members, but the full team discussed the results in several project meetings to ensure overall agreement. Criteria statements in the evaluation of indicators could also have been more specific and distinct to ensure meaningful responses. Most indicators were scored highly, and it is possible there was some confirmation or social desirability bias involved in these responses, given that assessors were involved in the original development of these indicators and responses were not anonymised. To mediate bias, assessors were requested to review the indicators based on their experiences with implementation, with the clear understanding that the indicators needed constructive feedback and revision. Resulting scores ranged widely—from three to 10—and text responses included constructive critique. The indicators were used for project monitoring by teams during the COVID-19 pandemic, which may have made the collection of information more challenging than it would have been otherwise, so the low feasibility rating of indicators may reflect some of these COVID-19 related challenges. Yet, we also recognise that WHO’s close collaboration with Ministries of Health may have facilitated access to information and without these relationships, feasibility scores may have been lower. Finally, while we consider the indicators to be applicable for a federalised system, they may require some adaptation in future work to be specified for each region or area.

The initial goal of this work was to monitor a health system strengthening project for quality abortion care, not to develop a normative set of indicators, and we do not intend for our indicators to be adopted verbatim elsewhere. In this paper, we present an initial attempt to develop indicators to measure health system capacity for quality abortion care, along with clear suggestions for how these indicators could be strengthened in future work. Our work had several strengths. The indicators, structure and criteria were developed by a group with broad knowledge in the relevant areas, were implemented as a baseline with two follow-up rounds, and were implemented in multiple countries with diverse contexts. Structured feedback was obtained from the experience of implementation. Most of the indicators we developed scored highly against predetermined criteria, and the indicators were drawn from existing data sources and aligned with existing health system monitoring efforts. These indicators and the future directions we have identified therefore fill a significant gap in abortion monitoring and evaluation and will serve as building blocks for future projects.

## Conclusion

Meeting Sustainable Development Goal targets 3.1, 3.7, 5.6 and other commitments to advance SRHR depends on evidence-based planning by health system leaders, policy-makers and others. Having identified a gap for indicators in health system capacity to provide quality abortion care, in this paper we shared a set of indicators developed for a multicountry, 4-year project focusing on health system strengthening for abortion. After multiple rounds of implementation, we have made progress toward identifying appropriate domains and indicators that are valid, feasible, useful and important. We have also identified challenges associated with these indicators and potential areas for strengthening. We have outlined further work that needs to be done in the area before a core set of indicators can be recommended. Establishing a core set of indicators to facilitate measurement of health system capacity for quality abortion care is critical for motivating action and accountability in reproductive health and rights and meeting Sustainable Development Goal commitments.

## supplementary material

10.1136/bmjph-2023-000401 online supplemental file 1

10.1136/bmjph-2023-000401 online supplemental file 2

10.1136/bmjph-2023-000401 online supplemental file 3

## Data Availability

Data are available upon reasonable request.
