# Supplementary material for: Development of indicators to measure health system capacity for quality abortion care in 10 countries: a rapid assessment of a measurement framework and indicators
Source: BMJ Public Health. 2024 May 6;2(1):e000401. doi: 10.1136/bmjph-2023-000401 (PMC11812777; doi:10.1136/bmjph-2023-000401)
Supplement: online supplemental file 1 [file bmjph-2-1-s001.pdf]

Supporting Country Strategies to Reduce Maternal Mortality and Achieve SDG Targets through a Health Systems Approach

Monitoring and Evaluation Framework * Indicator List

Supporting Country Strategies to Reduce Maternal Mortality and Achieve SDG Targets through a Health Systems Approach			
**Monitoring and Evaluation Framework**			
	**Domain**	**Indicator number**	**INDICATORS**
	**LEADERSHIP & GOVERNANCE**		
1	Level of political commitment to sexual and reproductive health and rights (SRHR), including abortion-related rights, through policies, strategies and approaches for health system governance	IND 1.1	Decreasing unsafe abortion is part of national strategy, plan or similar (for example for Maternal and Neonatal Health, Reproductive Health or similar area)
		IND 1.2	SRHR integrated into country cooperation strategy (CCS) and other relevant national strategic documents / roadmaps (e.g. UNDAF)
		IND 1.3	MoH has SRHR steering group or coordination mechanism operated with WHO participation and support
		IND 1.4	Protocols for comprehensive abortion care aligned with global standards are in national medical / treatment guidelines
		IND 1.5	Number of laws / policies / strategies / regulations / guidelines developed or updated in alignment with global or WHO SRHR guidelines
		IND 1.6	Descriptive assessment of extent to which tools and guidance to operationalize CAC enabling policies/guidelines etc. exist
	**HEALTH WORKFORCE**		
2	Extent to which systems are in place to support adequate production, availability and distribution of health workers trained in provision of abortion-related care	IND 2.1	Existence of institutional models for assessing and monitoring staffing needs for sexual and reproductive health service delivery
		IND 2.2	Proportion of accredited education institutions for all relevant cadres with a competency-based SRHR component in curricula (inclusive of SA/PAC/FP), consistent with global normative guidance
		IND 2.3	Number of graduates in past year from accredited education institutions with a competency-based SRHR component in curricula (inclusive of SA/PAC/FP), consistent with global normative guidance - for all relevant cadres
		IND 2.4	Country has system for in-service competency-based training in CAC, consistent with global normative guidance
		IND 2.5	Health workforce policies provide guidance to operationalize SRHR related priorities (e.g. urban-rural distribution, task sharing/skill mix, CHW utilization, etc.)
	**HEALTH INFORMATION**		
3	Inclusion of sexual and reproductive health indicators in the national health information system	IND 3.1	List of essential SRHR indicators, including SA/PAC/FP indicators, established within the national health system
		IND 3.2	Essential SRHR indicators, including SA/PAC/FP indicators, integrated into national health information system
		IND 3.3	DHIS2 module for SRHR, including SA/PAC/FP indicators, integrated into national HMIS.
		IND 3.4	Essential SRHR indicator data quality periodically assessed using WHO data quality review tools
		IND 3.5	CAC / PAC module integrated into the WHO health facility survey tool (HHFA or SARA) and/or other national monitoring platforms.
		IND 3.6	HMIS SRHR data, including data on SA/PAC/FP, used for planning, budgeting, or fundraising activities
	**MEDICINES & TECHNOLOGIES**		
4	Systems in place to promote availability of quality-assured essential abortion medicines	IND 4.1	National Essential Medicines List includes combination mifepristone and misoprostol, or misoprostol and mifepristone as separate presentations
		IND 4.2	Number of combination mifepristone and misoprostol and/or misoprostol and mifepristone as separate presentations submitted for market authorization, including through the WHO collaborative registration procedure for prequalified products
		IND 4.3	Number of MA products registered (combination mifepristone and misoprostol and / or misoprostol and mifepristone as separate presentations)
		IND 4.4	Pharmacovigilance system in place to monitor combination mifepristone and misoprostol and / or misoprostol and mifepristone as separate presentations
		IND 4.5	Combination mifepristone and misoprostol and/or misoprostol and mifepristone as separate presentations, are on national procurement lists, including tenders or other relevant documents
		IND 4.6	Combination mifepristone and misoprostol and/or misoprostol and mifepristone as separate presentations, procured in past 24 months via recognized procurement agents that serve the public sector
		IND 4.7	Forecasting tools for safe abortion essential medicines and products improved to align with national service capacity and to capture relevant information for national / regional market
		IND 4.8	Number of regulators participating in PQT trainings, observations, fellowships and other efforts
	**HEALTH FINANCING**		
5	Steps toward better tracking of financing flows allocated for SRH service provision, the level of inclusion into the benefits package of major national health financing mechanisms, and the way these mechanisms allocate resources to health service providers	IND 5.1	Essential SRH services have been assessed for inclusion in the Benefit Package as part of a systematic process including criteria on economic evidence and budget impact/costs
		IND 5.2	Number of health financing arrangements that have introduced new SRH essential services (including SA/PAC/FP) into their benefits package
		IND 5.3	Number of health financing instruments that have critically reviewed and adjusted their purchasing modalities – e.g. benefits specification including cost-sharing, payment methods, provider contracts –to boost service delivery of SRH essential services
		IND 5.4	Results of analysis of demand configuration and constraints to SRH essential services assessed and factored into health financing work
		IND 5.5	Public and external spending on reproductive health tracked

Indicator 1.1	**Decreasing unsafe abortion is part of a national strategy, plan or similar in Maternal and Neonatal Health, Reproductive Health or similar area**
Rationale	This indicator reflects state commitment to decreasing unsafe abortion and seeks to measure state recognition of the need to reduce unsafe abortion and state commitment to supporting national laws / policies / strategies / regulations / guidelines that promote decreasing unsafe abortion.
Definition	The indicator documents the existence of a national strategy or plan for maternal health, reproductive health, or similar, and whether decreasing unsafe abortion is included in the strategy.
Method of estimation/ calculation	Desk review
Numerator	Not applicable
Denominator	Not applicable
Preferred data sources	Nationally available official government document(s).<br>Collected specifically for the initiative.
Other possible data sources	
Disaggregation	None
Data type	Text
Data collection	Baseline, endline
Limitations	Regulatory change can be slow
Related indicators	
Related links	Safe abortion: technical and policy guidance for health systems<br>https://www.who.int/reproductivehealth/publications/unsafe_abortion/9789241548434/en/<br>Medical management of abortion:<br>https://www.who.int/reproductivehealth/guideline-medical-abortion-care/en/<br>Health worker roles in providing safe abortion care and post-abortion contraception:<br>https://apps.who.int/iris/bitstream/handle/10665/181041/9789241549264_eng.pdf?sequence=1

Indicator 1.2	**SRHR integrated into country cooperation strategy (CCS) and other relevant national strategic documents / roadmaps (e.g. UNDAF)**
Rationale	This indicator reflects state priorities and objectives in policies and strategies for health system governance towards an enabling SRHR environment. The indicator seeks to measure state recognition of the need to address SRHR and state commitment to supporting SRHR in national strategies.<br><br>WHO Country Cooperation Strategies (CCS) guide WHO's work in countries in terms of medium-term technical cooperation. In a a given Member State the CCS supports the country's national health policy, strategy or plan. The CCS is the basis for aligning WHO's collaboration with other United Nations bodies and development partners at the country level.<br><br>The United Nations Development Assistance Framework (UNDAF) is a strategic, medium term results framework that describes the collective vision and response of the UN system to national development priorities and results on the basis of normative programming principles.
Definition	The indicator documents whether SRHR is integrated into country cooperation strategy (CCS) and other relevant national strategic documents / roadmaps (e.g. UNDAF).
Method of estimation/ calculation	Desk review
Numerator	Not applicable
Denominator	Not applicable
Preferred data sources	Nationally available official government documents.<br>Collected specifically for the initiative.
Other possible data sources	
Disaggregation	None
Data type	Text
Data collection	Baseline, endline
Limitations	CCS are not updated frequently
Related indicators	
Related links	Safe abortion: technical and policy guidance for health systems<br>https://www.who.int/reproductivehealth/publications/unsafe_abortion/9789241548434/en/<br>Medical management of abortion:<br>https://www.who.int/reproductivehealth/guideline-medical-abortion-care/en/<br>Health worker roles in providing safe abortion care and post-abortion contraception:<br>https://apps.who.int/iris/bitstream/handle/10665/181041/9789241549264_eng.pdf?sequence=1

Indicator 1.3	**MoH has SRHR steering group or coordination mechanism operated with WHO participation and support**
Rationale	This indicator reflects MoH commitment to a coordinated approach to strengthening the SRHR enabling environment.
Definition	The indicator measures whether MoH has SRHR a steering group or coordination mechanism operated with WHO participation and support.
Method of estimation/ calculation	Desk review
Numerator	Not applicable
Denominator	Not applicable
Preferred data sources	Nationally available official government documents.<br>Collected specifically for the initiative.
Other possible data sources	
Disaggregation	None
Data type	Text
Data collection	Baseline, endline
Limitations	
Related indicators	
Related links	Safe abortion: technical and policy guidance for health systems<br>https://www.who.int/reproductivehealth/publications/unsafe_abortion/9789241548434/en/<br>Medical management of abortion:<br>https://www.who.int/reproductivehealth/guideline-medical-abortion-care/en/<br>Health worker roles in providing safe abortion care and post-abortion contraception:<br>https://apps.who.int/iris/bitstream/handle/10665/181041/9789241549264_eng.pdf?sequence=1

Indicator 1.4	**Protocols for comprehensive abortion care aligned with global standards are in national medical / treatment guidelines**
Rationale	This indicator reflects state commitment to meeting global standards of safe abortion care.
Definition	The indicator documents whether protocols for comprehensive abortion care aligned with global standards are included in national medical / treatment guidelines.
Method of estimation/ calculation	Desk review
Numerator	Not applicable
Denominator	Not applicable
Preferred data sources	Nationally available official government documents.<br>Collected specifically for the initiative.
Other possible data sources	
Disaggregation	None
Data type	Text
Data collection	Baseline, endline
Limitations	Regulatory change can be slow
Related indicators	
Related links	Safe abortion: technical and policy guidance for health systems<br>https://www.who.int/reproductivehealth/publications/unsafe_abortion/9789241548434/en/<br>Medical management of abortion:<br>https://www.who.int/reproductivehealth/guideline-medical-abortion-care/en/<br>Health worker roles in providing safe abortion care and post-abortion contraception:<br>https://apps.who.int/iris/bitstream/handle/10665/181041/9789241549264_eng.pdf?sequence=1

Indicator 1.5	**Number of laws / policies / strategies / regulations / guidelines developed or updated in alignment with global or WHO SRHR guidelines**
Rationale	This indicator reflects state priorities and objectives in policies and strategies for health system governance towards an enabling SRHR environment. The indicator seeks to measure the extent to which countries have national laws / policies / strategies / regulations / guidelines that promote an enabling environment for realizing SRHR.
Definition	The indicator documents the number of laws / policies / strategies / regulations / guidelines that have been developed or updated in alignment with global or WHO SRHR guidelines in the past
Method of estimation/ calculation	Count
Numerator	Not applicable
Denominator	Not applicable
Preferred data sources	Nationally available official government documents.<br>Collected specifically for the initiative.
Other possible data sources	
Disaggregation	None
Data type	Text
Data collection	Baseline, endline
Limitations	Regulatory change can be slow
Related indicators	
Related links	Safe abortion: technical and policy guidance for health systems<br>https://www.who.int/reproductivehealth/publications/unsafe_abortion/9789241548434/en/<br>Medical management of abortion:<br>https://www.who.int/reproductivehealth/guideline-medical-abortion-care/en/<br>Health worker roles in providing safe abortion care and post-abortion contraception:<br>https://apps.who.int/iris/bitstream/handle/10665/181041/9789241549264_eng.pdf?sequence=1

Indicator 1.6	**Descriptive assessment of extent to which tools and guidance exist to operationalize SRHR enabling policies.**
Rationale	This indicator reflects whether countries have appropriate codified approaches to enable users to transform enabling SRHR laws and policies into practice. The indicator seeks to measure intention in SRHR policies. Intention in SRHR policy formulation is reflected in instruments (i.e. governing tools, strategies, guidelines, health standards) facilitating SRHR policy implementation for example by addressing barriers to provision of care.
Definition	The indicator is a descriptive assessment of the extent to which tools and guidance to operationalize SRHR enabling policies/guidelines etc. exist.
Method of estimation/ calculation	Desk review
Numerator	Not applicable
Denominator	Not applicable
Preferred data sources	Nationally available official government documents.<br>Assessment conducted specifically for the initiative.
Other possible data sources	
Disaggregation	None
Data type	Text
Data collection	Baseline, endline
Limitations	Regulatory process change can be slow
Related indicators	1.5
Related links	Safe abortion: technical and policy guidance for health systems<br>https://www.who.int/reproductivehealth/publications/unsafe_abortion/9789241548434/en/<br>Medical management of abortion:<br>https://www.who.int/reproductivehealth/guideline-medical-abortion-care/en/<br>Health worker roles in providing safe abortion care and post-abortion contraception:<br>https://apps.who.int/iris/bitstream/handle/10665/181041/9789241549264_eng.pdf?sequence=1

Indicator 2.1	**Existence of institutional models for assessing and monitoring staffing needs for sexual and reproductive health service delivery (inclusive of SA/PAC/FP)**
Rationale	The ability of a country to meet its SRHR goals depends in part on adequate numbers of the people responsible for organizing and delivering health services. This indicator reflects the existance of a mechanism and/or responsible body in charge of determining the number of health workers of particular occupations required to effectively and safely deliver sexual and reproductive health services (inclusive of SA/PAC/FP) in health facilities and a mechanism to assess the sexual and reproductive health workload of health workers in health facilities.
Definition	The indicator documents the existence of institutional models for assessing and monitoring staffing needs for sexual and reproductive health service delivery (inclusive of SA/PAC/FP).
Method of estimation/ calculation	Desk review
Numerator	Not applicable
Denominator	Not applicable
Preferred data sources	Ministry of Health, regional ministries of health, insitutions or units responsible for policies on SRH workforce<br>Collected specifically for Initiative
Other possible data sources	Health facilities
Disaggregation	
Data type	
Data collection	Baseline, endline
Limitations	
Related indicators	
Related links	*WHONational health workforce accounts: A handbook*<br>https://apps.who.int/iris/bitstream/handle/10665/259360/9789241513111-eng.pdf?sequence=1

Indicator 2.2	**Proportion of accredited health education institutions for all relevant cadres with a competency-based SRHR component in curricula (inclusive of SA/PAC/FP), consistent with global normative guidance**
Rationale	This indicator reflects the proportion of accredited health education institutions for all relevant cadres with a competency-based SRHR component in the overall reproductive health curricula (inclusive of SA/PAC/FP), consistent with global normative guidance in terms of content and process. The ability of a country to meet its SRHR goals depends in part on the knowledge, skills and attitudes of the people responsible for organizing and delivering health services.<br><br>The indicator monitors availability of SRHR training at accredited health education institutions, by cadre being trained. Relevant cadre might include: pharmacists, doctors of complementary systems of medicine, auxiliary nurses, auxiliary nurse midwives, nurses, midwives, associate and advanced associate clinicians, non-specialist doctors, specialist doctors in obstetrics and gynecology and similar. The SA/PAC/FP component of a competency-based SRHR curricula should be consistent with global normative guidance and include training in contraception and abortion service delivery, counselling and provider–patient interaction, clinical skills, administrative/managerial issues and quality assurance.
Definition	The indicator documents the proportion of accredited health education institutions for all relevant cadres with a competency-based SRHR component in curricula (inclusive of SA/PAC/FP), consistent with global normative guidance
Method of estimation/ calculation	Proportion
Numerator	All accredited health education institutions nationally for health worker cadres engaged in providing SRH services with a competency-based SRHR component in the curricula (inclusive of SA/PAC/FP), consistent with global normative guidance
Denominator	All accredited health education institutions nationally for health worker cadres engaged in providing SRH services
Preferred data sources	National list of health educational institutions for each cadre, available curricula.<br>Ministry of Education, Ministry of Higher Education<br>Ministry of Labour and Human Resources<br>Collected specifically for Initiative
Other possible data sources	In some cases, data may be validated against registries of professional regulatory bodies where certification or licensure is required for practice.
Disaggregation	By cadre
Data type	
Data collection	Baseline, endline
Limitations	Curricula revision can be slow. There will be a need to make sure curricula revisions are holistic to the full scope of practice for each profession and not just one specific element
Related indicators	2.3
Related links	For further definition of health worker cadre, see WHO's *Health worker roles in providing safe abortion care and post-abortion contraception* :<br>https://apps.who.int/iris/bitstream/handle/10665/181041/9789241549264_eng.pdf?sequence=1<br><br>For global normative guidance, see<br>Safe abortion: technical and policy guidance for health systems<br>https://www.who.int/reproductivehealth/publications/unsafe_abortion/9789241548434/en/<br>Medical management of abortion:<br>https://www.who.int/reproductivehealth/guideline-medical-abortion-care/en/<br>Health worker roles in providing safe abortion care and post-abortion contraception:<br>https://apps.who.int/iris/bitstream/handle/10665/181041/9789241549264_eng.pdf?sequence=1

Indicator 2.3	**Number of graduates in past year from accredited health education institutions with a competency-based SRHR component in curricula (inclusive of SA/PAC/FP), consistent with global normative guidance - for all relevant cadres**
Rationale	This indicator reflects the quantity of graduates of all relevant cadres of accredited health education institutions with a competency-based SRHR component in the overall reproductive health curricula (inclusive of SA/PAC/FP), consistent with global normative guidance in terms of content and process. The ability of a country to meet its SRHR goals depends in part on the production of health cadre with the knowledge and skills required to organize and deliver health services.<br><br>The indicator supports monitoring of changes in the quantity of graduates of competency-based SRHR training at accredited training institutions, by cadre being trained. Relevant cadre include: pharmacists, doctors of complementary systems of medicine, auxiliary nurses, auxiliary nurse midwives, nurses, midwives, associate and advanced associate clinicians, non-specialist doctors, specialist doctors in obstetrics and gynecology and similar. The SA/PAC/FP component of a competency-based SRHR curricula should be consistent with global normative guidance and include training in the background for contraception and abortion service delivery, counselling and provider–patient interaction, clinical skills, administrative/managerial issues and quality assurance.
Definition	The indicator documents the number of graduates in the past year from accredited health education institutions with a competency-based SRHR component in curricula (inclusive of SA/PAC/FP), consistent with global normative guidance - for all relevant cadres.
Method of estimation/ calculation	Count
Numerator	Not applicable
Denominator	Not applicable
Preferred data sources	National list of educational institutions for each cadre, available curricula.<br>Ministry of Education, Ministry of Higher Education<br>Ministry of Labour and Human Resources<br>Databases on health education and training; health education and training institutions.<br>Collected specifically for Initiative
Other possible data sources	In some cases, data may be validated against registries of professional regulatory bodies where certification or licensure is required for practice.
Disaggregation	By cadre
Data type	
Data collection	Baseline, endline
Limitations	Curricula revision can be slow. There will be a need to make sure curricula revisions are holistic to the full scope of practice for each profession and not just one specific element
Related indicators	IND 2.2
Related links	For further definition of health worker cadre, see WHO's *Health worker roles in providing safe abortion care and post-abortion contraception* :<br>https://apps.who.int/iris/bitstream/handle/10665/181041/9789241549264_eng.pdf?sequence=1<br><br>For global normative guidance, see<br>Safe abortion: technical and policy guidance for health systems<br>https://www.who.int/reproductivehealth/publications/unsafe_abortion/9789241548434/en/<br>Medical management of abortion:<br>https://www.who.int/reproductivehealth/guideline-medical-abortion-care/en/<br>Health worker roles in providing safe abortion care and post-abortion contraception:<br>https://apps.who.int/iris/bitstream/handle/10665/181041/9789241549264_eng.pdf?sequence=1

Indicator 2.4	**Country has system for in-service competency-based training in CAC, consistent with global normative guidance**
Rationale	A system for in-service competency-based training in CAC indicates alignment of CAC in-service training with the broader health system. In-service CAC training is aimed at maintaining core competencies and developing new competencies to meet SRHR.
Definition	This indicator documents whether the country has a system for in-service competency-based training in CAC, consistent with global normative guidance.
Method of estimation/ calculation	Desk review
Numerator	Not applicable
Denominator	Not applicable
Preferred data sources	National health workforce policy, strategy and planning documents.<br>Collected specifically for the Initiative.
Other possible data sources	
Disaggregation	
Data type	
Data collection	Baseline, endline
Limitations	Curricula revision can be slow. There will be a need to make sure curricula revisions are holistic to the full scope of practice for each profession and not just one specific element
Related indicators	
Related links	For further definition of health worker cadre, see WHO's *Health worker roles in providing safe abortion care and post-abortion contraception* :<br>https://apps.who.int/iris/bitstream/handle/10665/181041/9789241549264_eng.pdf?sequence=1<br><br>For global normative guidance, see<br>Safe abortion: technical and policy guidance for health systems<br>https://www.who.int/reproductivehealth/publications/unsafe_abortion/9789241548434/en/<br>Medical management of abortion:<br>https://www.who.int/reproductivehealth/guideline-medical-abortion-care/en/<br>Health worker roles in providing safe abortion care and post-abortion contraception:<br>https://apps.who.int/iris/bitstream/handle/10665/181041/9789241549264_eng.pdf?sequence=1

Indicator 2.5	**Health workforce policies provide guidance to operationalize SRHR related priorities (e.g. urban-rural distribution, task sharing/skill mix, CHW utilization, etc.)**
Rationale	This indicator reflects whether countries have appropriate codified approaches to enable the transformation of enabling SRHR health workforce policies (e.g. urban-rural distribution, task sharing/skill mix, CHW utilization) into practice. Health workforce policy guidance on the organization and delivery of services influences the ability of a country to meet its SRHR goals.<br><br>The indicator seeks to measure intention in SRHR health workforce policies. Intention is reflected in instruments (i.e. governing tools, strategies, guidelines, health standards) supporting health workforce SRHR (inclusive of SA/PAC/FP) policy implementation.
Definition	The indicator documents whether health workforce policies provide guidance to operationalize SRHR related priorities (e.g. urban-rural distribution, task sharing/skill mix, CHW utilization, etc.).
Method of estimation/ calculation	Desk review
Numerator	Not applicable
Denominator	Not applicable
Preferred data sources	National health workforce policy instruments, including governing tools, strategies, guidelines, health standards, etc.<br>Collected specifically for the Initiative.
Other possible data sources	
Disaggregation	
Data type	
Data collection	Baseline, endline
Limitations	Policy change can be slow
Related indicators	
Related links	*WHO National health workforce accounts: A handbook*<br>https://apps.who.int/iris/bitstream/handle/10665/259360/9789241513111-eng.pdf?sequence=1<br>WHO's *Health worker roles in providing safe abortion care and post-abortion contraception* :<br>https://apps.who.int/iris/bitstream/handle/10665/181041/9789241549264_eng.pdf?sequence=1

Indicator 3.1	**List of essential SRHR indicators, including SA/PAC/FP indicators, established within the national health system**
Rationale	
Definition	The indicator measures whether a list of essential SRHR indicators, including SA/PAC/FP indicators, has been established within the national health system.
Method of estimation/ calculation	Desk review
Numerator	Not applicable
Denominator	Not applicable
Preferred data sources	Ministry of Health documents, including health information system documents.<br>Collected specifically for the Initiative.
Other possible data sources	
Disaggregation	
Data type	
Data collection	Baseline, endline
Limitations	
Related indicators	
Related links	https://sdg-tracker.org/gender-equality<br>https://www.thelancet.com/journals/lancet/article/PIIS0140-6736(18)30293-9/fulltext

Indicator 3.2	**Essential SRHR indicators, including SA/PAC/FP indicators, integrated into national health information system**
Rationale	Sound and reliable information on SRHR including safe abortion, postabortion, and contraception is the foundation of evidence-based decision-making in these areas and essential for evidence-based SRHR policy development and implementation, governance and regulation, health research, human resources development, health education and training, service delivery and financing.This indicator reflects whether nationally-identified SRHR essential indicators are included in data collection instruments supporting the national health information system, and reported in NHIS reports.
Definition	The indicator documents whether priority SRHR indicators, including SA/PAC/FP indicators are integrated into national health information system
Method of estimation/ calculation	Desk review
Numerator	Not applicable
Denominator	Not applicable
Preferred data sources	Data collection instruments and reporting from key information systems (CRVS, HMIS, MDSR, Survey),<br>Collected specifically for the initiative.
Other possible data sources	Health information system reports on SRHR services.
Disaggregation	By data sources
Data type	
Data collection	Baseline, endline
Limitations	Weak CRVS and HMIS, irregular population surveys
Related indicators	
Related links	Service availability and readiness assessment (SARA):<br>https://www.who.int/healthinfo/systems/sara_introduction/en/<br>Service Provision Assessment (SPA):<br>https://dhsprogram.com/What-We-Do/Survey-Types/SPA.cfm<br>Civil registration and vital statistics (CRVS):<br>https://www.who.int/healthinfo/civil_registration/en/<br>Maternal Death Surveillance and Response (MDSR)<br>https://www.who.int/maternal_child_adolescent/epidemiology/maternal-death-surveillance/en/

Indicator 3.3	**DHIS2 module for SRHR, including SA/PAC/FP indicators, integrated into national HMIS.**
Rationale	The District Health Information System 2 (DHIS2) open-source data collection and management tool provides users with a flexible interface for managing health data. The platform has data collection and management capabilities, built-in data validation, visualization and analysis tools, allowing end-users to access and analyse health data at all levels of the health system. Use of electronic forms for data collection supports more efficient and accurate collation of data at the national level with better quality control measures. Implementation of a DHIS2 module (standard configuration package) for SRHR, including SA/PAC/FP indicators, would facilitate SRHR data collection and reporting. Currently WHO has configuration packages in the health areas/programmes including EPI, HIV, Malaria and TB.
Definition	The indicator documents whether a DHIS2 module for SRHR, including SA/PAC/FP indicators, has been integrated into national HMIS
Method of estimation/ calculation	Desk review
Numerator	
Denominator	
Preferred data sources	Ministry of Health documents, including health information system documents.<br>Collected specifically for the Initiative.
Other possible data sources	
Disaggregation	
Data type	
Data collection	Baseline, endline
Limitations	Existence of DHIS2 package does not mean it will be used. Need country level support to promote use and effectiveness.
Related indicators	
Related links	https://who.dhis2.org/documentation/index.html#epi<br>https://www.who.int/healthinfo/tools_data_analysis_routine_facility/en/<br>https://www.who.int/bulletin/volumes/98/1/19-239970/en/

Indicator 3.4	**Essential SRHR indicator data quality periodically assessed using WHO Data Quality Review tools**
Rationale	Sound decisions for SRHR policy development and implementation, governance and regulation, health research, human resources development, health education and training, service delivery and financing are based on sound and reliable data. Therefore, it is essential to ensure that collected data are of good quality. WHO Data Quality Review tools support routine, annual and periodic independent assessments of facility-reported data. The tools are designed to assess the quality of data generated by information system(s) based in health facilities, and include guidelines and tools that lay the basis for a common understanding of data quality. In addition, they promote the institutionalization of data quality review in country. Guidelines and associated tools are organized into 3 distinct modules: (1) Framework and metrics; (2) Desk review of data quality; (3) Data verification and system assessment. Data quality metrics include: indicator-specific quality level (based on completeness, timeliness, consistency, reliability and accuracy) and overall composite score at the NHIS level.
Definition	The indicator documents whether essential SRHR indicator data quality is periodically assessed using WHO Data Quality Review tools
Method of estimation/ calculation	Desk review
Numerator	
Denominator	
Preferred data sources	Data quality review reports from assessments of SRHR data from key information systems (CRVS, HMIS, MDSR, Surveys).<br>Collected specifically for the initiative.
Other possible data sources	
Disaggregation	By data source and facility
Data type	
Data collection	Baseline, endline
Limitations	
Related indicators	
Related links	WHO Data Quality Review (DQR) Toolkit<br>https://www.who.int/healthinfo/tools_data_analysis/dqr_modules/en/

Indicator 3.5	**CAC / PAC module integrated into the WHO health facility survey tool (HHFA or SARA) and/or other national monitoring platforms.**
Rationale	Assessing the availability, readiness, safety and quality of Comprehensive Abortion Care (CAC) is not routinely done as part of the health care delivery system assessment, using facility survey such as the Harmonized Health Facility Assessment (HHFA), Service Availability and Readiness Assessment (SARA) or Service Provision Assessment (SPA) and other national health system monitoring programs. Data are essential to support the planning and management of a health systems. Inclusion of standardized indicators relating to abortion in national health system monitoring platforms will facilitate evidence-based health system planning and management.
Definition	This indicator measures whether abortion-related questions have been integrated into standardized health system data collection platforms including WHO's Harmonized Health Facility Assessment (HHFA), Service Availability and Readiness Assessment (SARA) and Service Provision Assessment (SPA) questionnaires, civil registration and vital statistics (CRVS), national health management information system (HMIS), and maternal death surveillance and response (MDSR) data collection.
Method of estimation/ calculation	Desk review of health facility survey tools (HHFA, SARA, CRVS, MDSR) and/or other national monitoring platforms (national HMIS) to document the inclusion status of a CAC/PAC module or abortion related questions.
Numerator	Not applicable
Denominator	Not applicable
Preferred data sources	Data collection instruments from key facility-based information systems (HHFA, SARA, SPA, CRVS, MDSR, HMIS).<br>Collected specifically for the initiative.
Other possible data sources	Reports on SRHR services
Disaggregation	
Data type	
Data collection	Baseline, endline
Limitations	
Related indicators	
Related links	Service Availability and Readiness Assessment (SARA) indicators and questionnaire:<br>https://www.who.int/healthinfo/systems/sara_indicators_questionnaire/en/<br>Civil registration and vital statistics (CRVS):<br>https://www.who.int/healthinfo/civil_registration/en/<br>Health management information system (HMIS):<br>https://www.who.int/healthinfo/tools_data_analysis_routine_facility/en/<br>Maternal death surveillance and response (MDSR) technical guidance:<br>https://www.who.int/maternal_child_adolescent/documents/maternal_death_surveillance/en/<br>Harmonized Health Facility Assessment (HHFA) - expected mid-2020

Indicator 3.6	**HMIS abortion-related SRHR, including CAC, data used for planning, budgeting, or fundraising activities**
Rationale	To implement programming to address abortion-related morbidity and mortality, decision makers need to be convinced of the need to address the issue and convinced that there are viable pathways for policy development and implementation. HMIS evidence supports the definition of the nature of the issues and their causes, cost-effective policy options, as well as key implementation considerations. HMIS-generated evidence supports the tracking of policy and program impact.
Definition	This indicator measures whether HMIS abortion-related SRHR data was used for planning, budgeting, or fundraising activities in past year
Method of estimation/ calculation	Desk review
Numerator	Not applicable
Denominator	Not applicable
Preferred data sources	Reports of planning, budgeting, or fundraising from past year,<br>Collected specifically for the initiative.
Other possible data sources	Policy briefs, reports of policy dialogues
Disaggregation	
Data type	
Data collection	Baseline, endline
Limitations	
Related indicators	
Related links	https://www.who.int/evidence/en/

Indicator 4.1	**National Essential Medicines List includes combination mifepristone and misoprostol, or misoprostol and mifepristone as separate presentations**
Rationale	This indicator reflects whether access to key SRH medicines mifepristone and misoprostol in combination, or misoprostol and mifepristone as single presentations are recognized as essential within the health care system. The national essential medicines list is a policy document that identifies medicines and health products considered to be essential to the functioning of the national health care system.
Definition	This indicator measures whether the national essential medicines list includes combination mifepristone and misoprostol, or misoprostol and mifepristone as separate presentations.
Method of estimation/ calculation	Desk review
Numerator	Not applicable
Denominator	Not applicable
Preferred data sources	National Essential Medicines List<br>Collected specifically for Initiative
Other possible data sources	
Disaggregation	
Data type	
Data collection	Baseline, endline
Limitations	
Related indicators	The indicator is reported in the Global Abortion Policies Database, supported by WHO and HRP
Related links	WHO Model Lists of Essential Medicines:<br>https://www.who.int/medicines/publications/essentialmedicines/en/

Indicator 4.2	**Number of combination mifepristone and misoprostol and/or misoprostol and mifepristone as separate presentations submitted for market authorization, including through the WHO collaborative registration procedure for prequalified products.**
Rationale	To gain market authorization an manufacturer or sponsor must submit detailed technical information, clinical study data, and other information as required by the NMRA and may also be required to submit to a factor inspection. This process is mandatory for all medicinal products. An increased number of submissions demonstates increased interest in marketing the product.
Definition	The indicator demonstrates whether or not mifepristone and misoprostol and/or misoprostol and mifepristone as separate presentations been submitted for market authorization, including through the WHO collaborative registration procedure for prequalified products.
Method of estimation/ calculation	Desk review
Numerator	Not applicable
Denominator	Not applicable
Preferred data sources	Information from National Medicines Regulatory Authority<br>Conducted specifically for the initiative
Other possible data sources	
Disaggregation	
Data type	
Data collection	Baseline, endline
Limitations	
Related indicators	4.3
Related links	Collaborative Procedure for Accelerated Registration:<br>https://extranet.who.int/prequal/content/collaborative-procedure-accelerated-registration

Indicator 4.3	**Number of MA products registered (combination mifepristone and misoprostol and / or misoprostol and mifepristone as separate presentations)**
Rationale	Registration of combination mifepristone and misoprostol and/or misoprostol and mifepristone as separate presentations (as finished pharmaceutical products (FPPs)).<br><br>Medicines registration is the process by which a national regulatory authority approves the use of a medicine in a country, having considered evidence of the Medicine's safety, quality and efficacy. It is thus primarily concerned with protecting public health.
Definition	The indicator measures how many combination mifepristone and misoprostol and / or misoprostol and mifepristone as separate presentations products have been registered.
Method of estimation/ calculation	Desk review
Numerator	Not applicable
Denominator	Not applicable
Preferred data sources	Information from National Medicines Regulatory Authority<br>Conducted specifically for the initiative
Other possible data sources	
Disaggregation	
Data type	
Data collection	Baseline, endline
Limitations	
Related indicators	4.2
Related links	Collaborative Procedure for Accelerated Registration:<br>https://extranet.who.int/prequal/content/collaborative-procedure-accelerated-registration

Indicator 4.4	**National pharmacovigilance system in place to monitor combination mifepristone and misoprostol and/ or misoprostol and mifepristone as separate presentations**
Rationale	This indicator measures whether a pharmacovigilance (PV) system for mifepristone and misoprostol is in place. A PV system for mifepristone and misoprostol aims to enhance patient care and patient safety in relation to the use of the medicines; and to support public health programmes by providing reliable, balanced information for the effective assessment of the risk-benefit profile of medicines. A PV system to monitor mifepristone and misoprostol includes activities to detect, assess, understand and prevent adverse effects or any other drug-related problem. The aims of the PV system are to enhance patient care and patient safety in relation to the use of mifepristone and misoprostol. Minimum Requirements for a Functional National Pharmacovigilance System<br><br>The following are the minimum requirements that the WHO and partners agree should be present in any national pharmacovigilance system.<br>1. A national pharmacovigilance centre with designated staff (at least one full time), stable basic funding, clear mandates, well defined structures and roles and collaborating with the WHO Programme for International Drug Monitoring.<br>2. The existence of a national spontaneous reporting system with a national individual case safety report (ICSR) form i.e. an ADR reporting form.<br>3. A national database or system for collating and managing ADR reports.<br>4. A national ADR or pharmacovigilance advisory committee able to provide technical assistance on causality assessment, risk assessment, risk management, case investigation and, where necessary, crisis management including crisis communication.
Definition	The indicator documents the existence of a national pharmacovigilance system to monitor combination mifepristone and misoprostol and/ or misoprostol and mifepristone as separate presentations.
Method of estimation/ calculation	Desk review
Numerator	Not applicable
Denominator	Not applicable
Preferred data sources	Reports from national pharmacovigilance centre, or similar government office responsible for pharmacovigilance<br>Collected specifically for initiative
Other possible data sources	
Disaggregation	
Data type	
Data collection	Baseline, endline
Limitations	Regulatory process change is slow and there will be a need to make sure such mechanisms are holistic to the full mifepristone and misoprostol pharmacovigilance system not just one specific element
Related indicators	
Related links	WHO Global Benchmarking Tool (GBT)<br>https://www.who.int/medicines/regulation/benchmarking_tool/en/<br>WHO Global Benchmarking Tool (GBT) For Evaluation of National Regulatory System of Medical Products: https://www.who.int/medicines/regulation/03_GBT_VL_RevVI.pdf?ua=1<br>Pharmacovigilance<br>https://www.who.int/medicines/areas/quality_safety/safety_efficacy/pharmvigi/en/<br>Minimum Requirements for a Functional National Pharmacovigilance System<br>https://www.who.int/medicines/areas/quality_safety/safety_efficacy/PV_Minimum_Requirements_2010_2.pdf

Indicator 4.5	**Combinations of mifepristone and misoprostol, including individual or combipack presentations are on national procurement lists, including tenders or other relevant documents**
Rationale	This indicator reflects whether or not the public sector has included mifepristone and misoprostol, including individual or combipack presentations on national procurement lists, including tenders or other relevant documents. Inclusion on the national procurement list serves as an indicator of product integration in the health system, including regular supply channels to health facilities and reimbursement schemes.
Definition	The indicator documents whether combinations of mifepristone and misoprostol, including individual or combipack presentations are on national procurement lists, including tenders or other relevant documents.
Method of estimation/ calculation	Desk review
Numerator	Not applicable
Denominator	Not applicable
Preferred data sources	National procurement authority interviews and records.<br>Conducted specifically for Initiative
Other possible data sources	
Disaggregation	
Data type	
Data collection	Baseline, endline
Limitations	
Related indicators	
Related links	

Indicator 4.6	**Combinations of mifepristone and misoprostol, including individual or combipack presentations, procured in past 24 months via recognized procurement agents that serve the public sector**
Rationale	This indicator reflects whether or not the public sector procures on a regular basis mifepristone and misoprostol, including individual or combipack presentations. Regular procurement serves as an indicator of product integration in the health system, including regular supply channels to health facilities and reimbursement schemes. Procurement should occur every two years or more frequently. If procurement cycles show longer times, it would suggest that product is not sufficiently available or may be expiring before use. If there is no evidence of procurement by a national authority or their officially designated proxy, it could imply lack of availability. In instances where product is available, but does not appear to be procured by the national authority or their delegate, it could imply information on importations, unstable supply and/or illegal distribution of the products.<br><br>Procurement for the purposes of this definition includes evidence that the products were included in national procurement tenders or contracts issued by the national procurement authority or their officially designated delegate. In the case where procurement is managed in a decentralized manner in the country, then the procurement should be documented through reviews of a sample of the highest levels of procurement authorities.
Definition	The indicator documents whether combinations of mifepristone and misoprostol, including individual or combipack presentations, have been procured in the past 24 months via recognized procurement agents that serve the public sector.
Method of estimation/ calculation	Desk review
Numerator	Not applicable
Denominator	Not applicable
Preferred data sources	National procurement authority interviews and records.<br>Conducted specifically for Initiative
Other possible data sources	
Disaggregation	
Data type	
Data collection	Baseline, endline
Limitations	
Related indicators	
Related links	

Indicator 4.7	**Forecasting tools for safe abortion essential medicines and products improved to align with national service capacity and to capture relevant information for the national / regional market.**
Rationale	Forecast information should form the basis of a procurement plan for the country. Effective procurement forecasting requires the following be considered:<br>- Available financing<br>- Stock on hand at all levels of distribution system<br>- Orders that are expected to be delivered<br>- Expected losses through expiry or damage<br>- Medicines donations<br>- Desired stock at end of each planning period (safety and working stock at all levels)
Definition	The indicator documents whether forecasting tools for safe abortion essential medicines and products have been improved to align with national service capacity and to capture relevant information for the national / regional market
Method of estimation/ calculation	Desk review
Numerator	Not applicable
Denominator	Not applicable
Preferred data sources	National procurement authority interviews and records.<br>Conducted specifically for Initiative
Other possible data sources	
Disaggregation	
Data type	
Data collection	Baseline, endline
Limitations	
Related indicators	
Related links	https://www.who.int/medicines/areas/access/supply/en/index2.html

Indicator 4.8	**Number of regulators participating in Prequalification Team (PQT) trainings, observations, fellowships and other efforts**
Rationale	The PQT trainings focus on the quality aspects of assessment of a product dossier as submitted to PQT for medicines, bioequivalence and biowaiver issues, as well as product information and labelling considerations. The trainings provide practical examples and solutions for common assessment issues. PQT training is aimed at regulators from national medicines regulatory authorities (NMRAs) in emerging markets, and primarily quality assessors.
Definition	This indicator documents the number of regulators participating in Prequalification Team (PQT) trainings, observations, fellowships and other efforts
Method of estimation/ calculation	Desk review
Numerator	Not applicable
Denominator	Not applicable
Preferred data sources	National medicines regulatory authority interviews and records.<br>Conducted specifically for Initiative
Other possible data sources	
Disaggregation	
Data type	
Data collection	Baseline, endline
Limitations	PQT trainings are coordinated by WHO headquarters and are implemented sporadically dependent on available funding.
Related indicators	This indicator is tracked by WHO Department of Regulation of Medicines and other Health Technologies
Related links	https://extranet.who.int/prequal/

Indicator 5.1	**Essential SRH services have been assessed for inclusion in the Benefit Package as part of a systematic process including criteria on economic evidence and budget impact/costs**
Rationale	One key objective is to make sure that decision making on whether an intervention should be added to the list of essential services (i.e. those which governments will at least partly co-finance) is grounded in evidence. One source of evidence should be economic analysis.
Definition	The indicator documents whether countries have assessed essential SRH services for inclusion in the Benefit Package as part of a systematic process including criteria on economic evidence and budget impact/costs.
Method of estimation/ calculation	Desk review of data use in the negotiations around the list of essential services or benefit packages (at scheme level)
Numerator	Not applicable
Denominator	Not applicable
Preferred data sources	Assessment of country production and use of economic analytical data to inform decision making around inclusion of SRH essential services in national essential service lists cofinanced by government or in benefit package of financing schemes<br>Conducted specifically for this Initiative
Other possible data sources	
Disaggregation	
Data type	Qualitative
Data collection	Baseline and endline
Limitations	
Related indicators	
Related links	Making fair choices on the path to universal health coverage:<br>https://www.who.int/choice/documents/making_fair_choices/en/

Indicator 5.2	**Number of health financing arrangements which have introduced new SRH essential services (including SA/PAC/FP) into their benefits package**
Rationale	A key policy instrument to improve financial protection for people accessing SRH services is to have these services covered by the existing health financing instruments in the country. This indicator reflects inclusion of SRH essential services into the benefits packages covered by health financing schemes (e.g. health insurance schemes, voucher programmes, etc.)<br>The indicator aims to track whether SRH essential are covered, and to provide detail on what is covered, for whom, where and how.
Definition	The indicator documents the number of health financing arrangements in the country which have introduced new SRH essential services (including SA/PAC/FP) into their benefits package
Method of estimation/ calculation	Desk review
Numerator	Not applicable
Denominator	Not applicable
Preferred data sources	Review of benefits packages and conditions for coverage of major health funding mechanisms in each country of the initiative<br>Collected specifically for the Initiative.
Other possible data sources	Policy documents/activity reports from major health financing schemes or programmes in a given country
Disaggregation	
Data type	
Data collection	Baseline and endline
Limitations	
Related indicators	
Related links	Benefits design:<br>https://apps.who.int/iris/bitstream/handle/10665/112671/9789241507158_eng.pdf?sequence=1

Indicator 5.3	**Number of health financing instruments which have critically reviewed and adjusted their purchasing modalities – e.g. benefits specification including cost-sharing, payment methods, provider contracts – to boost service delivery of SRH essential services**
Rationale	For health financing schemes to improve the coverage they provide for SRH essential services, they need to review their current purchasing arrangements for this set of services, and progressively move toward improved purchasing modalities.
Definition	This indicator consists of counting the number of health financing schemes that have conducted analysis to support decision making with regard to purchasing arrangements to cover SRH essential services.
Method of estimation/ calculation	Count
Numerator	Not applicable
Denominator	Not applicable
Preferred data sources	Assessment of health financing schemes' purchasing arrangements for SRH services in the country. Conducted specifically for the initiative
Other possible data sources	Policy documents/activity reports from major health financing schemes or programmes in a given country
Disaggregation	
Data type	
Data collection	Baseline and endline
Limitations	1. Acceptability of the scope of the work by health financing schemes or programmes<br>2. Access to data may be limited as data sets are not always public<br>3. Availability of data may be limited<br>4. Granularity of data may be limited
Related indicators	
Related links	

Indicator 5.4	**Results of analysis of demand configuration and constraints to SRH essential services assessed and factored into health financing work**
Rationale	The configuration of health financing is country specific, since the demography, epidemiology, culture and history, as well as spending requirements and available resources are different for every country.
Definition	This indicator documents that results of analysis of demand configuration and constraints to SRH essential services have been assessed and factored into health financing work.
Method of estimation/ calculation	Desk review
Numerator	Not applicable
Denominator	Not applicable
Preferred data sources	Assessment of health financing priority setting in the country.<br>Collected specifically for the initiative
Other possible data sources	Policy health financing documents/activity reports in a given country
Disaggregation	
Data type	
Data collection	Baseline and endline
Limitations	1. Acceptability of the scope of the work by health financing operators<br>2. Access to data may be limited as data sets are not always public<br>3. Availability of data may be limited<br>4. Granularity of data may be limited
Related indicators	
Related links	https://www.who.int/bulletin/volumes/94/6/15-155721/en/

Indicator 5.5	**Public and external spending on reproductive health tracked**
Rationale	Policy makers in Member States and stakeholders are progressively more aware of the value of tracking resources for health.<br><br>The WHO Health Accounts Country Platform provides countries with the framework, tools and technical support to institutionalize and set up a harmonized, integrated platform for annual and timely collection of health expenditure data. This serves to strengthen the capacity of the health account team in the country to report health expenditures using the global standard, the System of Health Accounts (SHA 2011), and to analyse and produce data relevant for national planning purposes.<br><br>Health accounts deliver means to learn retrospectively from past expenditure, improving planning and allocation of resources and increasing systems accountability. This aims to help member states protect their people from catastrophic health bills, reduce inequities in health and make definitive strides towards universal health coverage.<br>https://www.who.int/health-accounts/universal_health_coverage/en/
Definition	This indicator documents whether public and external spending on reproductive health is tracked
Method of estimation/ calculation	Desk review
Numerator	Not applicable
Denominator	Not applicable
Preferred data sources	WHO Health Accounts Country Platform (WHO/HQ)
Other possible data sources	Ministry of Health reports
Disaggregation	
Data type	
Data collection	Baseline, endline
Limitations	
Related indicators	This indicator is reported in WHO's global health financing reports
Related links	https://www.who.int/health-accounts/universal_health_coverage/en/<br>https://www.who.int/health_financing/strategy/revenue_collecton/en/
