# Supplementary material for: Development of indicators to measure health system capacity for quality abortion care in 10 countries: a rapid assessment of a measurement framework and indicators
Source: BMJ Public Health. 2024 May 6;2(1):e000401. doi: 10.1136/bmjph-2023-000401 (PMC11812777; doi:10.1136/bmjph-2023-000401)
Supplement: online supplemental file 2 [file bmjph-2-1-s002.pdf]

SRHR INITIATIVE MONITORING DATA COLLECTION TOOL

SRHR INITIATIVE BASELINE TOOL

COUNTRY:

REGION:

DATE COMPLETED:

NAME OF OFFICER RESPONSIBLE FOR COMPLETING DATA COLLECTION:

	Question Number	Question			Comments
**SECTION 1 LEADERSHIP AND GOVERNANCE**					
1.1	1.1.1	Are there national policies/strategies on reproductive health care?	YES		
			NO	skip to 1.2.1	
	1.1.2	Do these policies/strategies on reproductive health care include components on safe abortion care?	YES		
		If yes, please file the national policy(ies)/strategy(ies) that refer(s) to safe abortion care.	NO		
	1.1.3	Do these policies/strategies on reproductive health care include components on postabortion care?	YES		
		If yes, please file the national policy(ies)/strategy(ies) that refer(s) to postabortion care.	NO	skip to 1.2.1	
	1.1.4	Is/Are copy/(ies) of the national policy(ies)/strategy(ies) that refer to decreasing unsafe abortion filed in the appropriate baseline folder?	YES		
			NO		
1.2	1.2.1	Is SRHR integrated into the country cooperation strategy (CCS) and other relevant national strategic documents / roadmaps (e.g. United Nations Development Assistance Framework (UNDAF))?	YES		
		If yes, for CCS and each additional national strategic document that has integrated SRHR, please file document referring to strengthening the SRHR enabling environment.	NO	skip to 1.3.1	
	1.2.2	Are texts of the country cooperation strategy (CCS) and other relevant national strategic documents that have integrated SRHR filed in the appropriate baseline folder?	YES		
			NO		
1.3	1.3.1	Does MoH have SRHR steering group or coordination mechanism?	YES		
		If yes, please file the text of the ToR and membership of the SRHR steering group or coordination mechanism.	NO	skip to 1.4.1	
	1.3.2	Is the MoH SRHR steering group or coordination mechanism operated with WHO participation?	YES		
			NO		
	1.3.3	*Is a copy of the ToR and membership of the SRHR steering group or coordination mechanism filed in the appropriate baseline folder?*	YES		
			NO		
1.4	1.4.1	Have national protocols for delivery of postabortion care been developed?	YES		
		If yes, please file the text of the text of the protocols.	NO	skip to 1.4.4	
	1.4.2	Are protocols in national medical/treatment guidelines for postabortion care aligned with current WHO or other global standards?	YES		
			NO		
	1.4.3	Are protocols in national medical/treatment guidelines for postabortion care - aligned with current WHO or other global standards - filed in the appropriate baseline folder?	YES		
			NO		
	1.4.4	Have national protocols for delivery of abortion care been developed?	YES		
		If yes, please file the text of the text of the protocols.	NO	skip to 1.5.1	
	1.4.5	Are protocols in national medical/treatment guidelines for abortion care aligned with current WHO or other global standards?	YES		
			NO		
	1.4.6	Are protocols in national medical/treatment guidelines for abortion care filed in the appropriate baseline folder?	YES		
			NO		
1.5	1.5.1	Please complete Baseline Table 1.5 (see Tab). From Table 1.5, column 1 assess the number of national laws / policies / strategies / regulations / guidelines referencing safe abortion care, postabortion care and contraceptive care.			
		write number in box ->			
1.6	1.6.1	Describe extent to which tools and guidance (i.e. governing tools, strategies, guidelines, health standards) exist to operationalize safe abortion care service delivery alignment with current global standards.	Click here for options		
		Click text box for options: high / medium / minimal or none			
	1.6.2	Describe extent to which tools and guidance (i.e. governing tools, strategies, guidelines, health standards) exist to operationalize care for abortion complications (PAC) service delivery alignment with current global standards.	Click here for options		
		Click text box for options: high / medium / minimal or none			
	1.6.3	Describe extent to which tools and guidance (i.e. governing tools, strategies, guidelines, health standards) exist to operationalize contraceptive / FP service delivery alignment with current global standards.	Click here for options		
		Click text box for options: high / medium / minimal or none			

SRHR INITIATIVE MONITORING DATA COLLECTION TOOL

SECTION 2 HEALTH WORKFORCE

2.1	2.1.1	Is there a mechanism and/or responsible body in charge of determining the number of health workers of particular occupations required to effectively and safely deliver sexual and reproductive health services in health facilities?	YES		
			NO	skip to 2.1.3	
	2.1.2	Please provide the name of the mechanism and/or responsible body in charge of determining the number of health workers of particular occupations required to effectively and safely deliver sexual and reproductive health services (inclusive of SA/PAC/FP) in health facilities.			
	2.1.3	Is there a mechanism to assess the sexual and reproductive health workload of health workers in health facilities?	YES		
			NO	skip to 2.2.1	
	2.1.4	Please provide the name of the mechanism to assess the sexual and reproductive health workload of health workers in health facilities.			
2.2	2.2.1	In column 1, below, list all health worker cadres engaged in providing SRH services.<br>For each health worker cadre listed, complete a copy of Table 2.2 (see Tab)<br>Use results of Table 2.2 to complete columns 2 and 3, in the table below.<br>Please use additional rows if required.	Number of education institutions for each specified cadre	% of education institutions* with SA/PAC/FP component in curricula	

1	2	3
1		
2		
3		
4		
5		
6		
7		
8		
9		
10		
11		
12		
13		
14		
15		

**In countries with over 30 health education institutions per cadre, select a representative sample of leading health education institutions. See instructions in Table 2.2.*

2.3	2.3.1	In Table 2.2 column 7 (see tab), for each health worker cadre, indicate the number of graduates per year from each specified institution having received education in SA and/or PAC and/or FP, consistent with global normative guidance			
2.4	2.4.1	Complete table 2.4 (see tab) about in-service competency-based training in safe abortion care, postabortion care and contraceptive care, consistent with current WHO or other global normative guidance.			
	2.5.1	Do health workforce policies provide guidance to operationalize SRHR related priorities (e.g. urban-rural distribution, task sharing/skill mix, CHW utilization,	YES		
		If yes, please file text of policy(ies).	NO	skip to 3.1.1	
	2.5.2	Is/are the document(s) filed in the appropriate Baseline folder?	YES		
			NO		
	2.5.3	Describe extent to which health workforce policies provide guidance to operationalize SRHR-related priorities (e.g. urban-rural distribution, task<br>*Click text box for options: high / medium / minimal or none*	Click here for options		
2.5					

SRHR INITIATIVE MONITORING DATA COLLECTION TOOL

	Question Number	Question			Comments
**SECTION 3 HEALTH INFORMATION**					
	3.1.1	Has the national health system established a national list of essential/priority SRHR indicators?	YES	Go to Table 3.0, Q 3.1.1	
		If yes, please please file text of the national list of essential/priority SRHR indicators established by national health system.	NO	Skip to 3.2.1	
	3.1.2	Is/are the document(s) filed in the appropriate baseline folder?	YES		
3.1			NO		
	3.2.1	Are SRHR indicators, including SA and/or PAC and/or FP indicators, integrated into the national health information system?	YES	Go to Table 3.0, Q 3.2.1	
		If yes, please please file the text of instruments from the national health information system that include national essential/priority SRHR indicators, including SA/PAC/FP indicators.	NO	Skip to 3.3.1	
	3.2.2	Is/are the document(s) filed in the appropriate baseline folder?	YES		
3.2			NO		
	3.3.1	Does the country use a DHIS2 platform for the national HMIS?	YES		
			NO	Skip to 3.4.1	
		Is a DHIS2 module for SRHR integrated into the national HMIS?	YES	Go to Table 3.0, Q 3.3.1	
		If yes, please please file text of DHIS2 module for SRHR.	NO	Skip to 3.4.1	
	3.3.2	Is the document filed in the appropriate baseline folder?	YES		
3.3			NO		
	3.4.1	Is data quality for SRHR indicators periodically assessed using WHO data quality review tools?	YES	Go to Table 3.0, Q 3.4.1	
		If yes, please file the most recent data quality report for SRHR indicators.	NO	Skip to 3.5.1	
	3.4.2	Is the document filed in the appropriate baseline folder?	YES		
3.4			NO		
	3.5.1	Are SA and/or PAC and/or FP questions integrated into the WHO health facility assessment tool (SARA or HHFA) and/or other national monitoring platforms?	YES	Go to Table 3.0, Q 3.5.1	
		If yes, please file report of WHO health facility assessment (SARA or HHFA) and/or other national monitoring platforms that include ***SA and/or PAC and/or FP***	NO	Skip to 3.6.1	
	3.5.2	Is/are the document(s) filed in the appropriate baseline folder?	YES		
3.5			NO		
	3.6.1	Has HMIS abortion-related SRHR data been used for planning, budgeting, or fundraising activities in the past year?	YES	Go to Table 3.0, Q 3.6.1	
		If yes, please file key documents from the past year showing HMIS abortion and contraceptive related SRHR data being used for planning, budgeting, or fundraising activities.	NO	Skip to 4.1.1	
	3.6.2	Is/are the document(s) filed in the appropriate baseline folder?	YES		
3.6			NO		

SRHR INITIATIVE MONITORING DATA COLLECTION TOOL

	Question Number	Question			Comments
**SECTION 4 MEDICINES & TECHNOLOGIES**					
	4.1.1	Does the national Essential Medicines List include combination mifepristone and misoprostol?	YES		
			NO	Skip to 4.1.3	
	4.1.2	For what indication(s) is combination mifepristone and misoprostol on the national Essential Medicines List?			
	i				
	ii				
	iii				
	iv				
	4.1.3	Does the national Essential Medicines List include misoprostol?	YES		
			NO	Skip to 4.1.5	
	4.1.4	For what indication(s) is misoprostol on the national Essential Medicines List?			
	i				
	ii				
	iii				
	iv				
	4.1.5	Does the national Essential Medicines List include mifepristone?	YES		
			NO	Skip to 4.1.7	
	4.1.6	For what indication(s) is mifepristone on the national Essential Medicines List?			
	i				
	ii				
	iii				
	iv				
	4.1.7	*Please file National Essential Medicines List including combination mifepristone and misoprostol, or misoprostol and mifepristone as separate presentations.*	YES		
4.1		Is the national EML filed in the appropriate baseline folder?	NO		
	4.2.1	How many **combination mifepristone and misoprostol products** have been submitted for market authorization, including through the WHO collaborative registration procedure for prequalified products?			
		write number in box ->			
	4.2.2	How many **misoprostol products** (as separate presentations) have been submitted for market authorization, including through the WHO collaborative registration procedure for prequalified products?			
		write number in box ->			
	4.2.3	How many **mifepristone products** (as separate presentations) have been submitted for market authorization, including through the WHO collaborative registration procedure for prequalified products?			
4.2		*write number in box ->*			
	4.3.1	Does **combination mifepristone and misoprostol** have market authorization with the National Medicines Regulatory Authority (NMRA), including through the WHO collaborative registration procedure for prequalified products?	YES		
			NO		
	4.3.2	How many **combination mifepristone and misoprostol products** have been registered?			
		write number in box ->			
	4.3.3	Does **misoprostol** (as a separate presentation) already have market authorization with the National Medicines Regulatory Authority (NMRA), including through the WHO collaborative registration procedure for prequalified products?	YES		
			NO		
	4.3.4	How many **misoprostol products** (as separate presentations) have been registered?			
		write number in box ->			
	4.3.5	Does m**ifepristone** (as a separate presentation) already have market authorization with the National Medicines Regulatory Authority (NMRA), including through the WHO collaborative registration procedure for prequalified products?	YES		
			NO		
	4.3.6	How many **mifepristone products** (as separate presentations) have been registered?			
4.3		*write number in box ->*			
	4.4.1	Is a national pharmacovigilance system in place to monitor combination mifepristone and misoprostol?	YES		
			NO		
	4.4.2	Is a national pharmacovigilance system in place to monitor misoprostol as a separate presentation?	YES		
			NO		
	4.4.3	Is a national pharmacovigilance system in place to monitor mifepristone as a separate presentation?	YES		
4.4			NO		
	4.5.1	Are combinations of mifepristone and misoprostol, including individual or combipack presentations, on national procurement lists, including tenders or other relevant documents?	YES		
4.5			NO		
	4.6.1	Have combinations of mifepristone and misoprostol, including individual or combipack presentations, been procured in past 24 months via recognized procurement agents that serve the public sector?	YES		
4.6			NO		
	4.7.1	Do forecasting tools for safe abortion essential medicines and products align with national service capacity to capture relevant information for the national/regional market?	YES		
4.7			NO		
	4.8.1	How many regulators have participated in Prequalification Team (PQT) trainings, observations, fellowships and other efforts in past year?			
4.8		*write number in box ->*			

SRHR INITIATIVE MONITORING DATA COLLECTION TOOL

	Question Number	Question			Comments
**SECTION 5 HEALTH FINANCING**					
	5.1.1	Have essential SRH services been assessed for inclusion in the national Benefit Package as part of a systematic process including criteria on economic evidence and budget impact/costs?	YES		
			NO		
	5.1.2	*Please file document(s) on assessment of SRH services for inclusion in the national Benefit Package, including criteria on economic evidence and budget impact/costs.*	YES		
5.1		Is/are the document(s) filed in the appropriate baseline folder?	NO		
	5.2.1	Complete table 5.2 indicating which of the major health financing schemes in the country have SRH essential services (including SA and/or PAC and/or FP) in their benefits package.			
5.2		*Note: A "major" health financing scheme is defined as one that has national coverage.*			
	5.3.1	Complete table 5.3 indicating which of the major health financing schemes have critically assessed and adjusted - if necessary - their purchasing modalities to boost service delivery of SRH essential services.			
5.3	5.3.2	In table 5.3 for each major health financing scheme that has carried out such an assessment, please provide a short summary of the assessment and the subsequent actions taken.			
	5.4.1	Have barriers to key populations accessing essential SRH services been assessed in past 5 years?	YES		
		If yes, please file any assessment report(s) if available.	NO	Skip to 5.4.3	
	5.4.2	Is /Are the report(s) filed in the appropriate baseline folder?	YES		
			NO		
	5.4.3	Were results of the SRH barriers assessment shared with the major health financing schemes?	YES		
5.4			NO		
	5.5.1	Is public and external spending on reproductive health tracked?	YES		
5.5			NO		
