# Supplementary material for: Development of indicators to measure health system capacity for quality abortion care in 10 countries: a rapid assessment of a measurement framework and indicators
Source: BMJ Public Health. 2024 May 6;2(1):e000401. doi: 10.1136/bmjph-2023-000401 (PMC11812777; doi:10.1136/bmjph-2023-000401)
Supplement: online supplemental file 3 [file bmjph-2-1-s003.pdf]

SUPPORTING COUNTRY STRATEGIES TO REDUCE MATERNAL MORTALITY AND ACHIEVE SDG TARGETS THROUGH A HEALTH SYSTEMS APPROACH

MONITORING AND EVALUATION

The initiative monitoring framework was developed to fill a gap in monitoring health system capacity in the area of abortion care. Initiative indicators were developed to align with the WHO health system building blocks; to draw on available existing health system information; to provide inputs useful for program planning and improvement; and as a whole set of indicators, to represent health system capacity to prevent unsafe abortion.

There is a growing interest within and outside of WHO in accessing and using the Initiative indicators. We have an opportunity to share the framework, the indicators and our experiences using these.

*

INSTRUCTIONS FOR PROVIDING FEEDBACK ON INITIATIVE MONITORING FRAMEWORK

To assess the monitoring framework as a whole and the individual indicators, information on the user experience is essential. The worksheet below was developed to allow quick sharing of experience with each indicator in each health system building block area, and provide more details as necessary. Please reflect on the questions below and record your reflections in the worksheet. Question 1 relates to column 1, Question 2 relates to worksheet column 2, etc. Each country and regional office represented in the Initiative TWG is requested to complete a worksheet.

Questions for reflection: Initiative monitoring framework

For worksheet column 1:

1. Did the indicator make sense? Was the definition clear? Did the indicator apply in context? (Please respond YES or NO in worksheet response column 1).
    a. If not, please describe what did not make sense and/or suggest an improvement (provide feedback in column 5)

For worksheet column 2:

2. Was the information collected accessible / easy to collect once the correct source was identified? (Please respond YES or NO in worksheet response column 2).
    a. If not, how could it be improved? (provide feedback in column 5)

For worksheet column 3:

3. Was the information collected useful for program planning or program improvement? (Please respond YES or NO in worksheet response column 3).
    a. If yes, how so? (provide feedback in column 5)
    b. If not, how could it be improved? (provide feedback in column 5)

For worksheet column 4:

4. Does the information represent an important component of health system capacity to prevent unsafe abortion / provide safe abortion? (Please respond YES or NO in worksheet response column 4; provide additional feedback in column 5)

For worksheet column 5:

5. Where you have additional experiences and opinions to share, please share these in column 5.

Final question:

6. Considering the entire set of indicators, do you feel the information collected - when compiled - reflects key structures and inputs required for service delivery, without actually measuring service delivery, outcomes and impact? If not, what should be modified to improve the set of indicators? (Provide feedback at end row of worksheet).

Initiative monitoring feedback worksheet

Indicator		1<br>Makes sense<br>(Yes/No)	2<br>Information accessible<br>(Yes/No)	3<br>Useful for program planning<br>(Yes/No)	4<br>Important<br>(Yes/No)	5<br>Additional feedback
1	**Leadership and governance**					
1.1	Decreasing unsafe abortion is part of national strategy, plan or similar (for example for Maternal and Neonatal Health, Reproductive Health or similar area)					
1.2	SRHR integrated into country cooperation strategy (CCS) and other relevant national strategic documents / roadmaps (e.g. UNDAF)					
1.3	MoH has SRHR steering group or coordination mechanism operated with WHO participation and support					
1.4	Protocols for comprehensive abortion care aligned with global standards are in national medical / treatment guidelines					
1.5	Number of laws / policies / strategies / regulations / guidelines developed or updated in alignment with global or WHO SRHR guidelines					
1.6	Descriptive assessment of extent to which tools and guidance to operationalize CAC enabling policies/guidelines etc. exist					
Do you have any additional comments to share on individual leadership and governance indicators or the set of indicators?						

Indicator		1 Makes sense (Yes/No)	2 Information accessible (Yes/No)	3 Useful for program planning (Yes/No)	4 Important (Yes/No)	5 Additional feedback
**2**	**Health workforce**					
**2.1**	Existence of institutional models for assessing and monitoring staffing needs for sexual and reproductive health service delivery					
**2.2**	Proportion of accredited education institutions for all relevant cadres with a competency-based SRHR component in curricula (inclusive of SA/PAC/FP), consistent with global normative guidance					
**2.3**	Number of graduates in past year from accredited education institutions with a competency-based SRHR component in curricula (inclusive of SA/PAC/FP), consistent with global normative guidance - for all relevant cadres					
**2.4**	Country has system for in-service competency-based training in CAC, consistent with global normative guidance					
**2.5**	Health workforce policies provide guidance to operationalize SRHR related priorities (e.g. urban-rural distribution, task sharing/skill mix, CHW utilization, etc.)					
Do you have any additional comments to share on individual health workforce indicators or the set of indicators?						

Indicator		1<br>Makes sense<br>(Yes/No)	2<br>Information accessible<br>(Yes/No)	3<br>Useful for program planning<br>(Yes/No)	4<br>Important<br>(Yes/No)	5<br>Additional feedback
**3**	**Health information**					
**3.1**	List of essential SRHR indicators, including SA/PAC/FP indicators, established within the national health system					
**3.2**	Essential SRHR indicators, including SA/PAC/FP indicators, integrated into national health information system					
**3.3**	DHIS2 module for SRHR, including SA/PAC/FP indicators, integrated into national HMIS.					
**3.4**	Essential SRHR indicator data quality periodically assessed using WHO data quality review tools					
**3.5**	CAC / PAC module integrated into the WHO health facility survey tool (HHFA or SARA) and/or other national monitoring platforms.					
**3.6**	HMIS SRHR data, including data on SA/PAC/FP, used for planning, budgeting, or fundraising activities					
Do you have any additional comments to share on individual health information indicators or the set of indicators?						

Indicator		1<br>Makes sense<br><br>(Yes/No)	2<br>Information accessible<br><br>(Yes/No)	3<br>Useful for program planning<br>(Yes/No)	4<br>Important<br><br>(Yes/No)	5<br>Additional feedback
**4**	**Medicines and Technologies**					
**4.1**	National Essential Medicines List includes combination mifepristone and misoprostol, or misoprostol and mifepristone as separate presentations					
**4.2**	Number of combination mifepristone and misoprostol and/or misoprostol and mifepristone as separate presentations submitted for market authorization, including through the WHO collaborative registration procedure for prequalified products					
**4.3**	Number of MA products registered (combination mifepristone and misoprostol and / or misoprostol and mifepristone as separate presentations)					
**4.4**	Pharmacovigilance system in place to monitor combination mifepristone and misoprostol and / or misoprostol and mifepristone as separate presentations					
**4.5**	Combination mifepristone and misoprostol and/or misoprostol and mifepristone as separate presentations, are on national procurement lists, including tenders or other relevant documents					
**4.6**	Combination mifepristone and misoprostol and/or misoprostol and mifepristone as separate presentations, procured in past 24 months via recognized procurement agents that serve the public sector					
**4.7**	Forecasting tools for safe abortion essential medicines and products improved to align with national service capacity and to capture relevant information for national / regional market					
**4.8**	Number of regulators participating in PQT trainings, observations, fellowships and other efforts					
Do you have any additional comments to share on individual medicines and technologies indicators or the set of indicators?						

	Indicator	1<br>Makes sense<br><br>(Yes/No)	2<br>Information accessible<br><br>(Yes/No)	3<br>Useful for program planning<br>(Yes/No)	4<br>Important<br><br>(Yes/No)	5<br>Additional feedback
**5**	**Health financing**					
**5.1**	Essential SRH services have been assessed for inclusion in the Benefit Package as part of a systematic process including criteria on economic evidence and budget impact/costs					
**5.2**	Number of health financing arrangements that have introduced new SRH essential services (including SA/PAC/FP) into their benefits package					
**5.3**	Number of health financing instruments that have critically reviewed and adjusted their purchasing modalities – e.g. benefits specification including cost-sharing, payment methods, provider contracts –to boost service delivery of SRH essential services					
**5.4**	Results of analysis of demand configuration and constraints to SRH essential services assessed and factored into health financing work					
**5.5**	Public and external spending on reproductive health tracked					
Do you have any additional comments to share on individual health financing indicators or the set of indicators?						

Indicator		1<br>Makes sense<br><br>(Yes/No)	2<br>Information accessible<br><br>(Yes/No)	3<br>Useful for program planning<br>(Yes/No)	4<br>Important<br><br>(Yes/No)	5<br>Additional feedback
**6**	**Service Delivery**					
6.1	Minimum Initial Services Package (MISP), inclusive of SA/PAC/FP, included in the national emergency preparedness plan					
The initiative monitoring framework was not structured to measure service delivery, as the initiative was meant to target the health system building blocks on which quality services rest. Given this, do you have any additional comments to share on the service delivery indicator or the indicator area?						
**ENTIRE SET OF INDICATORS (question 6)**<br>Considering the entire set of indicators, do you feel the information collected - when compiled - can indicate health system capacity to prevent unsafe abortion at a structural and input level? If not, what should be modified to improve the set of indicators?						
